# Canagliflozin mitigates ferroptosis and ameliorates heart failure in rats with preserved ejection fraction

**DOI:** 10.1007/s00210-022-02243-1

**Published:** 2022-04-27

**Authors:** Sai Ma, Li-Li He, Guo-Rui Zhang, Qing-Juan Zuo, Zhong-Li Wang, Jian-Long Zhai, Ting-Ting Zhang, Yan Wang, Hui-Juan Ma, Yi-Fang Guo

**Affiliations:** 1 Department of Internal Medicine, Hebei Medical University, Shijiazhuang, Hebei China; 2 Department of Internal Medicine, Hebei General Hospital, Shijiazhuang, Hebei China; 3 Department of Geriatric Cardiology, Hebei General Hospital, Shijiazhuang, Hebei China; 4 Department of Cardiology, The Third Hospital of Shijiazhuang City Affiliated to Hebei Medical University, Shijiazhuang, Hebei China; 5 Department of Physical Examination Center, Hebei General Hospital, Shijiazhuang, Hebei China; 6 Department of Cardiology, Hebei General Hospital, Shijiazhuang, Hebei China; 7 Department of Endocrinology, Hebei General Hospital, Shijiazhuang, Hebei China

**Keywords:** Canagliflozin, Sodium-glucose cotransporter 2 inhibitor, Ferroptosis, Lipid peroxidation, Heart failure

## Abstract

Recently, hypoglycemic drugs belonging to sodium-glucose cotransporter 2 inhibitors (SGLT2i) have generated significant interest due to their clear cardiovascular benefits for heart failure with preserved ejection fraction (HFpEF) since there are no effective drugs that may improve clinical outcomes for these patients over a prolonged period. But, the underlying mechanisms remain unclear, particularly its effects on ferroptosis, a newly defined mechanism of iron-dependent non-apoptotic cell death during heart failure (HF). Here, with proteomics, we demonstrated that ferroptosis might be a key mechanism in a rat model of high-salt diet-induced HFpEF, characterized by iron overloading and lipid peroxidation, which was blocked following treatment with canagliflozin. Data are available via ProteomeXchange with identifier PXD029031. The ferroptosis was evaluated with the levels of acyl-CoA synthetase long-chain family member 4, glutathione peroxidase 4, ferritin heavy chain 1, transferrin receptor, Ferroportin 1, iron, glutathione, malondialdehyde, and 4-hydroxy-trans-2-nonenal. These findings highlight the fact that targeting ferroptosis may serve as a cardioprotective strategy for HFpEF prevention and suggest that canagliflozin may exert its cardiovascular benefits partly via its mitigation of ferroptosis.

**Supplementary Information:**

The online version contains supplementary material available at 10.1007/s00210-022-02243-1.

## Introduction

Chronic heart failure (CHF) is characterized by high mortality and morbidity rates and characterizes the end stages of various cardiovascular diseases (Kaspar et al. 2018). Between 40 and 60% of all HF cases are classified as preserved ejection fraction (HFpEF). Surprisingly, the currently applied drug treatments for HF with reduced ejection fraction (HFrEF) do not work in patients with HFpEF. In addition, despite considerable effort, there are still no long-term therapeutic treatments for HFpEF, with current therapies being insufficient to improve the clinical presentation and prognosis of HFpEF patients (Borlaug 2020). This means that novel treatments for this type of HF are needed with some urgency.

Sodium-glucose cotransporter 2 inhibitors (SGLT2i), a new class of hypoglycemic drugs, inhibit the activity of sodium-glucose cotransporter 2, which is involved in glucose re-absorption by proximal tubules (Kanai et al. 1994; Jhund et al. 2021; Špinar et al. 2021) and has attracted significant attention because of its protective effects on the cardiovascular system in both clinical and basic research settings. Several large randomized controlled trials (EMPA-REG, CANVAS, DELCARE TIMI-58, DAPA-HF, EMPEROR-Reduced, etc.) have confirmed that treatment with SGLT2i may significantly reduce the risk of cardiovascular and/or HF death and the occurrence of major adverse cardiovascular events for patients with coronary artery disease, with and without diabetes (Baker et al. 2014). In addition, the EMPEROR-Preserved study for HFpEF patients recently reported that empagliflozin treatment results in a significant reduction in the occurrence of the main composite endpoints for this condition (cardiovascular death and hospitalization due to HF) (Anker et al. 2019). This is a major milestone that may help usher in a new era for HFpEF treatment, but the mechanism underlying these effects is still not fully understood. We designed this study to explore the mechanism underlying the cardiovascular benefits of SGLT2i in HFpEF using Dahl salt-sensitive (DSS) rats exposed to a high-salt diet inducing hypertension and the classic HFpEF model (Nagae et al. 2009). We then treated these animals with canagliflozin, a representative drug of SGLT2i, and evaluated its effects in vivo.

Ferroptosis, a novel type of programmed cell death, was first proposed in 2012 and defined as cell death resulting from the iron-dependent generation of lipid peroxides and a significant increase in reactive oxygen species (ROS). This mechanism was shown to be distinct from apoptosis, necrosis, autophagy, and other cell death modalities, and is characterized by plasma membrane blistering, mitochondrial shrinkage, cristae reduction or disappearance, and an increase in membrane density. Biochemically, ferroptosis manifests as accumulated iron and oxygen-free radicals and depleted glutathione (Dixon et al. 2012). Accumulating evidence indicates that ferroptosis plays an important role in cardiovascular disease (Park et al. 2019; Tadokoro et al. 2020; Ni et al. 2021), renal failure (Friedmann Angeli et al. 2014), and several other conditions (Liang et al. 2019; Derry et al. 2020). A bioinformatic analysis validated that ceRNA regulatory networks were bridged with ferroptosis in HF (Zheng et al. 2021). Previous studies have confirmed that ferroptosis plays an important role in HfrEF (Liu et al. 2018; Wang et al. 2020a, b; Ning et al. 2021), but rare evidence shows how it affects HFpEF. Therefore, this study was designed to explore whether ferroptosis plays an important role in HFpEF rats and whether SGLT2i can improve HFpEF by regulating these effects. Our data may provide novel insights into the prevention and treatment of HFpEF.

## Materials and methods

### Reagents

Canagliflozin was purchased from Janssen-Cilag International NV (Xian, China). The malondialdehyde (MDA) and glutathione (GSH) assay kits were from Nanjing Jiancheng Bioengineering Institute (Nanjing, China), and the iron assay kit was from Sigma Aldrich Inc. (St. Louis, MO, USA). The Prussian blue iron staining assay kit was from Slolarbio (Beijing, China). The plasma brain natriuretic peptide (BNP) enzyme-linked immunosorbent assay (ELISA) kit was obtained from Cusabio (Wuhan, China), while the Insulin ELISA kit was obtained from ExCell Biology (Shanghai, China). One Step TUNEL Apoptosis Assay Kit was obtained from Beyotime Biotechnology (Nantong, China). The primary antibody against Ferroportin 1 (FPN1) was purchased from Bioss (bs-4906R, Beijing, China), while those against glutathione peroxidase 4 (GPX4) (ab125066), acyl-CoA synthetase long-chain family member 4 (ACSL4) (ab155282), ferritin heavy chain 1(FTH1) (ab183781), and transferrin Receptor (TFR1) (ab269514) were from Abcam (Cambridge, UK). The primary antibody against SLC7A11 (xCT) was purchased from Affinity Bioscience (DF12509, Jingsu, China). The primary antibody against 4-hydroxy-trans-2-nonenal (4-HNE) was from GeneTex (GTX01087, Southern California, USA) and those for nicotinamide adenine dinucleotide phosphate oxidase 4 (Nox4) (380,874), and β-actin (380,624) was from Zen-bioscience (Chengdu, China). Finally, the TRNzol Universal Reagent, FastKing RT Kit (With gDNase), and SuperReal PreMix Plus (SYBR Green) were purchased from TIANGEN (Beijing, China).

### Animal preparations

Experiments involving animals were approved by the Animal Care and Management Committee of Hebei General Hospital (permit number SYXK(JI)2015–0065) and performed in accordance with the ARRIVE guidelines 2.0 (Sert et al. 2020). Male DSS rats (body mass 200.00–270.00 g) (permit number SCXK (JING) 2016–0006) were purchased from Vital River, Beijing, China, at between 7 and 8 weeks of age and kept on a 12–12 h cycle with free access to water and food in the Clinical Research Center of the Hebei General Hospital. After a week of adaptive feeding, the rats were randomly assigned and the experiments were conducted using the following three groups for a period of 12 weeks: (1) normal group (*n* = 12): DSS rats received a low-salt diet (AIN-76A + 0.3% NaCl with irradiation, research diets) and received intragastric administration of vehicle HPMC (hydroxypropyl methylcellulose) (0.5%) (2 ml/kg/day), (2) HFpEF group (*n* = 12): DSS rats received a high-salt diet (AIN-76A + 8% NaCl with irradiation) and received intragastric administration of vehicle HPMC (0.5%) (2 ml/kg/day), and (3) Canagliflozin group (*n* = 12): DSS rats received a high-salt diet (AIN-76A + 8% NaCl with irradiation) and received intragastric administration of canagliflozin (20 mg/kg/day) dissolved in vehicle HPMC (0.5%) (2 ml/kg/day). The group size was based on the trials of previous animal studies (Fang et al. 2019).

### Blood pressure measurement

The blood pressure of experimental rats was measured weekly by tail-cuff plethysmography (BP-2000; Visitech Systems, Inc.) and recorded for 15 min continuously at the same time of the day. The data recorded were averaged from 6 effective values.

### General condition

The body weights were estimated weekly. At the 10th week of the experiment, metabolic cages (SA104, Jiangsu SANS Biological Technology Co. Ltd., China) were used to monitor food intake, water intake, and urine volume for 24 h.

### Echocardiography

At the end of the 12th week, transthoracic echocardiography was performed using a Vevo® 2100 Imaging System (FUJIFILM VisualSonics Inc., Toronto, Canada) under isoflurane-based anesthesia and M-mode and two-dimensional pulse-wave Doppler images were obtained. All measurements were performed by two experienced technicians blinded to the experimental group of the subject animals and the data are reported as the averages from three consecutive cycles per loop.

### Anesthesia

When rats in the HFpEF group achieved HFpEF criteria (Ho et al. 2020), specimens were collected after all rats being deeply isoflurane-based anesthetized. Under the premise of ensuring the normal operation of the anesthesia machine and the accessory connection system, the induced concentration of isoflurane was regulated at 3–4% and the anesthesia lasted about 2–3 min. Whole blood was collected from the abdominal aorta and centrifuged at 3000 RPM for 10 min to extract serum. Left ventricular (LV) weight and tibial length were measured.

### TMT-based proteomic analysis

The proteomics experiment and the targeted nano-LC­MS/MS analysis of LV apical tissue sampled from three different groups were performed. Each group had three mixed samples. After a series of steps, such as sample lysis, BCA assay, acetone precipitation, protein alkylation, protein digestion, TMT labeling, SDC cleanup, peptide desalting, and high­pH pre­fractionationin, peptides were collected, separated, and analyzed with a nano­UPLC (EASY­nLC1200) coupled to a Q Exactive HFX Orbitrap instrument (Thermo Fisher Scientific) with a nano­electrospray ion source. The vendor’s raw MS files were processed using MaxQuant software (Version 1.6.15.0). After the completion of the database search, the polypeptides and proteins matching to decoy database were filtered out. The screening criteria for these proteins were unique peptide ≥ 1, *P*-value < 0.05 and fold change < 0.83 or fold change > 1.2. Then, we mapped the genes to nodes in the gene ontology (GO) database for functional enrichment analysis and analyzed the metabolic pathways that were significantly enriched between the HFpEF group and the Cana group. The mass spectrometry proteomics data have been deposited to the ProteomeXchange Consortium via the PRIDE partner repository with the dataset identifier PXD029031 (Perez-Riverol et al. 2019).

### Biochemical analysis

Total cholesterol (TC), triglyceride (TG), low-density lipoprotein cholesterol (LDL-C), high-density lipoprotein cholesterol (HDL-C), and the relative concentrations of 3-hydroxybutyric acid (D3-H), creatinine, iron, MDA, and GSH in the sera from each group were assessed by the Nanjing Jiancheng Bioengineering Institute with automatic biochemical analyzer (DS-261, SINNOWA Nanjing Center, China). BNP and insulin levels in serum were determined using the appropriate ELISA kit. Iron (Fe^2+^) within the cardiac tissues was reacted with a chromogen substrate to produce a colorimetric (593 nm) change and reported as ng/mg of heart tissue. The MDA concentration was measured using the thiobarbituric acid method and monitored at 532 nm. GSH was reacted with dithio-dinitrobenzoic acid and evaluated by colorimetry at 405 nm. The MDA and GSH content were then reported as nanomole per milligram protein and micromole per gram protein.

### Histological analysis

Heart sections were stained with hematoxylin and eosin or Masson’s trichrome stain and observed under a microscope (Nikon Eclipse Ci-L, Japan) where we measured the myocyte diameters and interstitial LV fibrosis for each group using Image Pro Plus v6.0 software (Media Cybernetics, Inc.).

### Prussian blue iron staining

Myocardial slices were incubated with Prussian blue iron staining, then rinsed, dehydrated, sealed, and observed under the microscope.

### Immunohistochemical staining and immunofluorescence staining

After pretreatment, sections were incubated overnight with primary antibody against 4-HNE and TFR1, and then incubated with the corresponding secondary antibody. The histochemical images were obtained under the microscope and immunofluorescence staining was observed using a fluorescence microscope (Zeiss Imager.D2, Germany). Depositions of 4-HNE and TFR1 were analyzed by Image-Pro Plus 6.0 system software.

### TUNEL staining

The slices were added with 20 μg/ml DNase-free protease K drops, washed with PBS for three times, mixed with TUNEL solution, and then incubated at 37 °C for 60 min away from light. The tablets were sealed with an anti-fluorescence quenching solution and observed under a fluorescence microscope. The excitation wavelength range is 450–500 nm and the emission wavelength range is 515–565 nm (green fluorescence).

### Transmission electron microscopy (TEM)

To observe myocardial cell microstructure, two DSS rats in each group were randomly selected, and their tissues were fixed, hydrated, polymerized, cut into slices, double stained, and finally photographed using a HITACHI H-7650 TEM.

### Western blotting

After the total protein was extracted, the protein concentration was examined. With 50 μg of total protein, each protein sample was separated for electrophoresis on 8–12% sodium dodecyl sulfate–polyacrylamide gels and transferred onto polyvinylidene difluoride membranes. Then, the membranes would be blocked for 2 h at room temperature and incubated with the primary antibody against FPN1 (bs-4906R, Bioss, China), GPX4 (ab125066, Abcam, UK), ACSL4 (ab155282, Abcam, UK), FTH1 (ab183781, Abcam, UK), TFR1 (ab269514, Abcam, UK), xCT (DF12509, Affinity Bioscience, China), 4-HNE (GTX01087, GeneTex, USA), Nox4 (380,874, Zen-bioscience, China), and β-actin (380,624, Zen-bioscience, China) at 4 °C overnight. Then, the membranes were incubated with the second antibodies (Zen-bioscience, China) for 1 h at room temperature. The target bands were visualized by enhanced chemiluminescence and analyzed on Chemiluminescent gel imaging (MiniChemi 610 Plus, Beijing Saizhi Venture Technology Co., Ltd.), which were then quantified by ImageJ analysis software (National Institutes of Health, Bethesda, MD).

### RT-qPCR

Total RNA was extracted, reverse-transcribed, and used as the template in the RT-qPCR assays performed using ABI Prism v2.04 (Applied Biosystems, Foster City, CA, USA) on ABI 7500 PCR (Applied Biosystems). The specific primers used in these evaluations are listed in Table 1. Relative mRNA expression for the genes of interest was established using their 2 − ΔΔCt values. Each sample was run in triplicate, and the mean value of each set of triplicates normalized to that of rat β-actin.

**Table 1:** Primer sequence for RT-qPCR

Gene	Forward sequence (5’–3’)	Reverse sequence (5’–3’)	PL*
xCT	GGTGGTGTGTTTGCTGTCT	AGAGGAGTGTGCTTGTGGA	101
TFR1	CGGCTACCTGGGCTATTGTA	TTCTGACTTGTCCGCCTCTT	85
FTH1	GGCTGAATGCAATGGAGTGT	TCTTGCGTAAGTTGGTCACG	186
FPN1	TCCTGGGCTTCGACTGTATC	CAAGTGAAGGCCACAGTTCC	124
GPX4	AATTCGCAGCCAAGGACATC	GGCCAGGATTCGTAAACCAC	170
ACSL4	AGACAAACCCGGAAGTCCAT	AGGCTGTCCTTCTTCCCAAA	132
NOX4	GTGAACGCCCTGAACTTCTC	ATACCACCACCATGCAGACA	139
β-actin	CACCATGTACCCAGGCATTG	CCTGCTTGCTGATCCACATC	20

^*^PL, product length/bp

### Statistical analysis

Results are expressed as the mean ± standard deviation and were analyzed using SPSS software, version 17.0 (IBM, Inc.). Statistical comparisons were performed using one-way ANOVA followed by Bonferroni’s multiple comparison test or Tamhane’s multiple comparison test. The post hoc tests were done only if *F* is significant and there is no variance inhomogeneity. A *P*-value of < 0.05 was considered statistically significant.

## Results

### Effects of canagliflozin on the characteristics and biochemical indicators for HF in rats

Table 2 summarizes the basic characteristics and biochemical indicators of HF in each of the three groups. Rats in the hypertensive groups (HFpEF and Cana) consumed more chow and water than those in the control but had lower body weight with increased urine output. This was particularly marked in the Cana group. Despite this, there were no significant differences in the plasma concentrations of glucose, TG, TC, LDL-C, HDL-C, or Insulin between any of these groups. However, the high-salt diet DSS rats did demonstrate an increase in the plasma concentration of creatinine, BNP, and LV weight when indexed to tibial length, which were then reduced in the group receiving canagliflozin. Besides, canagliflozin increased the concentration of D3-H in the serum compared with the HFpEF group.

**Table 2:** Animal characteristics and cardiovascular biochemical indicators of rats at the 12th week

	Normal	HFpEF	Cana	*P*
	*n* = 12	*n* = 8	*n* = 12	
Body weight (g)	384.33 ± 20.94	356.70 ± 22.50*	330.16 ± 18.33*^#^	< 0.05
LV weight/TL (mg/mm)	20.14 ± 2.19	24.04 ± 2.69*	20.67 ± 2.52^#^	0.004
Creatinine (μmol/l)	27.53 ± 3.48	41.37 ± 5.25*	32.70 ± 2.90*^#^	< 0.05
Blood glucose (mmol/l)	4.6 ± 0.7	4.6 ± 0.5	4.1 ± 0.6	0.106
D3-H (mmol/l)	1.05 ± 0.07	1.06 ± 0.05	1.34 ± 0.10^#^	< 0.05
TC (mmol/l)	2.83 ± 0.32	3.18 ± 0.99	3.17 ± 0.33	0.335
TG (mmol/l)	0.49 ± 0.13	0.55 ± 0.22	0.50 ± 0.14	0.627
LDL-c (mmol/l)	1.58 ± 0.28	1.81 ± 0.23	1.76 ± 0.42	0.252
HDL-c (mmol/l)	0.55 ± 0.11	0.63 ± 0.13	0.52 ± 0.08	0.098
Insulin (μg/l)	0.33 ± 0.07	0.30 ± 0.02	0.30 ± 0.03	0.257
BNP (pg/ml)	201.96 ± 49.66	338.65 ± 23.33*	184.79 ± 56.12^#^	< 0.05
	*n* = 6	*n* = 6	*n* = 6	
Food intake (g/24 h)	15.22 ± 1.45	20.97 ± 1.20*	25.16 ± 4.46*^#^	< 0.05
Water intake (ml/24 h)	19.5 ± 7.9	82.8 ± 20.1*	135.2 ± 20.5*^#^	< 0.05
Urine volume (ml/24 h)	9.6 ± 4.4	57.2 ± 20.7*	102.7 ± 5.4*^#^	< 0.05

Data are presented as mean ± standard deviation. *p < 0.05 versus normal group. ^#^*p* < 0.05 versus HFpEF group. *LV weight/TL*, left ventricular weight/tibial length; *D3-H*, 3-hydroxybutyric acid; *TC*, total cholesterol; *TG*, triglyceride; *LDL-C*, low-density lipoprotein cholesterol; *HDL-C*, high-density lipoprotein cholesterol; *BNP*, brain natriuretic peptide

### Canagliflozin treatment improved blood pressure, cardiac remodeling, and function in HFpEF rats

**Fig. 1:**
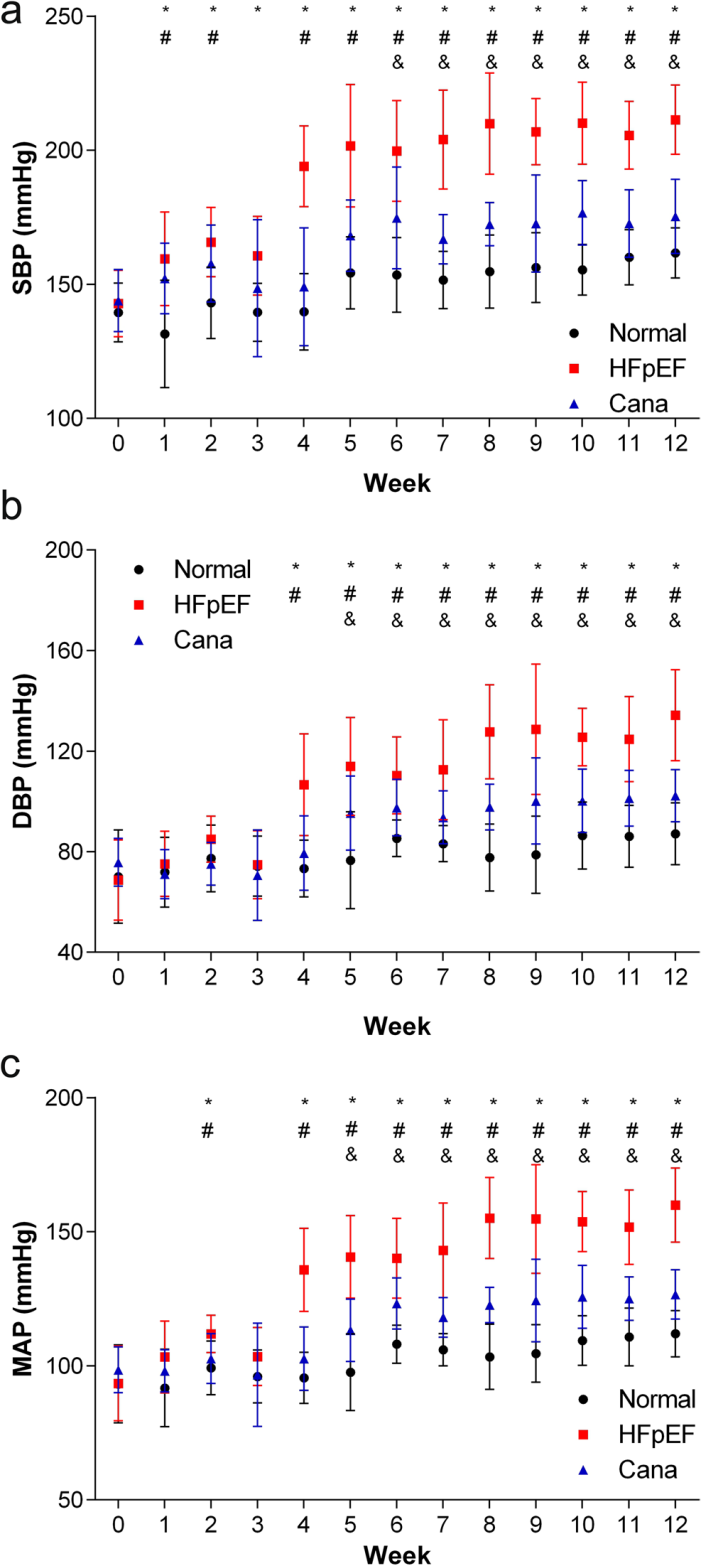
Serial measurements of blood pressure in rats. **a** SBP; **b** DBP; **c** MAP. *n* = 12 rats/group. Data are presented as mean ± standard deviation. *p < 0.05 HFpEF group versus normal group. #*p* < 0.05 Cana group versus HFpEF group. &*p* < 0.05 Cana group versus normal group. SBP, systolic blood pressure; DBP, diastolic blood pressure; MAP, mean arterial pressure

We also noted that the systolic pressure (SBP) (Fig. 1a), the diastolic blood pressure (DBP) (Fig. 1b), and the mean arterial pressure (MAP) (Fig. 1c) in the HFpEF group were markedly increased following four weeks of high-salt diet when compared to their starting values and those of the Cana and normal groups at the same time point, and this trend was maintained until the end of the study (Fig. 1). However, the SBP in the Cana group did not increase obviously but the DBP was significantly higher than that of the baseline after the 6th week of treatment, suggesting that canagliflozin may prevent the development of hypertension in HFpEF rats, especially for systolic blood pressure.

**Fig. 2:**
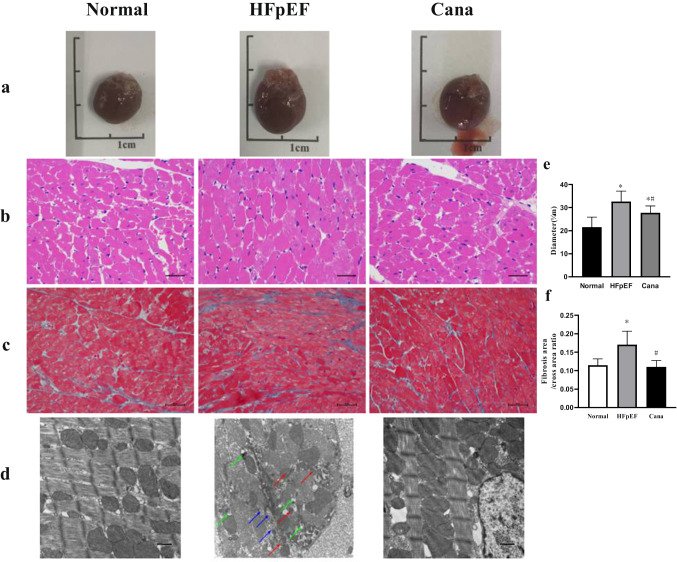
Gross hearts and myocardium structure in rats. **a**–**d** Gross hearts and representative HE, Masson’s trichrome, and TEM images are shown to visualize myocardium structure. **e**–**f** Canagliflozin reduced cardiomyocyte size and interstitial fibrosis in HFpEF rats. *n* = 6 rats/group. Data are presented as mean ± standard deviation. *p < 0.05 versus normal group. #*p* < 0.05 versus HFpEF group

**Fig. 3:**
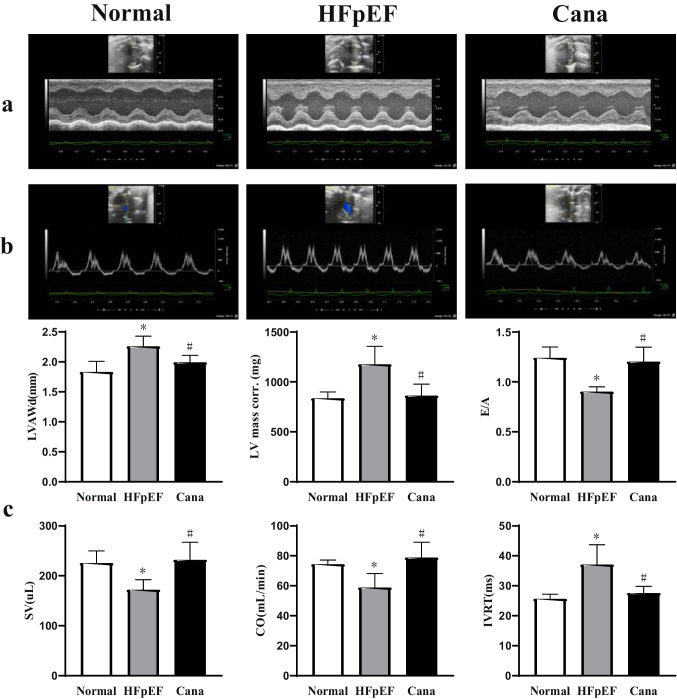
Echocardiographic assessment of LV structural and functional data in rats. **a**–**b** Echocardiographic representative M-mode and pulse-wave Doppler images to visualize myocardium structure. **c** Canagliflozin mitigated LV function in HFpEF rats indicated by LVAWd, LV mass corr., E/A, SV, CO, and IVRT. *n* = 6 rats/group. Data are presented as mean ± standard deviation. *p < 0.05 versus normal group. #*p* < 0.05 versus HFpEF group. LVAWd, left ventricular anterior wall in diastole; LV mass corr., left ventricular mass corrected; E/A, early diastolic filling to atrial filling velocity ratio of mitral flow; SV, stroke volume; CO, cardiac output; IVRT, isovolumic relaxation time

Rats in the HFpEF group demonstrated increased cardiomyocyte size and LV interstitial fibrosis when compared with the animals in the Normal group, and all of these values were returned almost to baseline when evaluated in the Cana group (Fig. 2). TEM images (Fig. 2d) revealed that most of the striated muscle was ruptured, and the Z-line and M-line could not be clearly distinguished in the HFpEF group samples. The red arrow represented mitochondria with thickened membranes and reduced cristae. The green arrow represented mitochondria with swollen appearance and even absent cristae partly. These were ferroptosis-specific characteristics. The blue arrow represented autophagosome production. However, canagliflozin treatment led to improved striated muscle arrangement, improved mitochondria performance, and reduced mitochondrial atrophy. We then explored the structural alterations using several key parameters measured by echocardiography (Fig. 3, Table 3). Representative images show that rats in the HFpEF group experienced preserved EF with increased wall thickness, LV mass, decreased E/A, stroke volume (SV), cardiac output (CO), and prolonged isovolumic relaxation time (IVRT) when compared with those of the Normal group. But, these changes were reversed in the Cana group. Besides, heart rate, left ventricular anterior wall in systole, left ventricular posterior wall in diastole, left ventricular posterior wall in systole, left ventricular internal diameter in diastole, left ventricular internal dimension in systole, ejection fraction, and fractional shortening did not change obviously. Taken together, this data suggests that canagliflozin may alleviate myocardial hypertrophy, interstitial fibrosis, and improve LV diastolic function in HFpEF rats.

**Table 3:** Canagliflozin mitigated LV function in HFpEF rats

	Normal	HFpEF	Cana	*P*
	*n* = 6	*n* = 6	*n* = 6	
HR (bpm)	349 ± 27	340 ± 23	353 ± 15	0.598
LVAWd (mm)	1.83 ± 0.18	2.26 ± 0.17*	1.99 ± 0.12^#^	0.001
LVAWs (mm)	3.27 ± 0.23	3.47 ± 0.20	3.44 ± 0.15	0.211
LVPWd (mm)	2.01 ± 0.23	2.26 ± 0.25	2.11 ± 0.23	0.208
LVPWs (mm)	3.34 ± 0.33	3.49 ± 0.21	3.46 ± 0.16	0.954
LVIDd (mm)	7.28 ± 0.34	7.09 ± 0.18	6.93 ± 0.73	0.446
LVIDs (mm)	3.40 ± 0.11	3.67 ± 0.28	3.62 ± 0.50	0.354
EF (%)	81 ± 5	80 ± 5	78 ± 4	0.530
FS (%)	51 ± 5	51 ± 5	48 ± 4	0.433
LV mass corr. (mg)	834.73 ± 64.32	1174.78 ± 181.82*	860.88 ± 116.47^#^	0.001
E/A	1.24 ± 0.11	0.90 ± 0.05*	1.20 ± 0.15^#^	< 0.05
SV (μL)	225.28 ± 24.58	171.83 ± 20.28*	231.66 ± 35.52^#^	0.003
CO (ml/min)	74.33 ± 2.82	58.70 ± 9.42*	78.71 ± 10.39^#^	0.015
IVRT (ms)	25.61 ± 1.56	37.00 ± 6.67*	27.51 ± 2.32^#^	0.009

Data are presented as mean ± standard deviation. *p < 0.05 versus normal group. #*p* < 0.05 versus HFpEF group. *HR*, heart rate; *LVAWd*, left ventricular anterior wall in diastole; *LVAWs*, left ventricular anterior wall in systole; *LVPWd*, left ventricular posterior wall in diastole; *LVPWs*, left ventricular posterior wall in systole; *LVIDd*, left ventricular internal diameter in diastole; *LVIDs*, left ventricular internal dimension in systole; *EF*, ejection fraction; *FS*, fractional shortening; *LV mass corr.*, left ventricular mass corrected; *E/A*, early diastolic filling to atrial filling velocity ratio of mitral flow; *SV*, stroke volume; *CO*, cardiac output; *IVRT*, isovolumic relaxation time

### TMT-based proteomic analysis

**Fig. 4:**
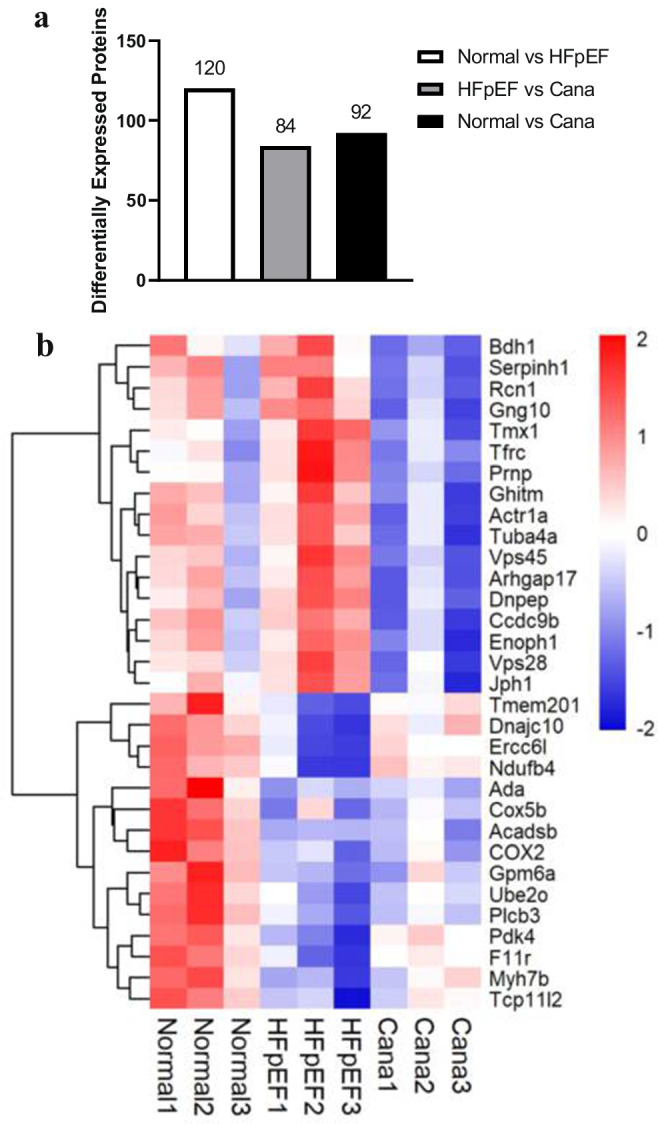
Proteomics was performed in cardiac tissues. **a** The column diagram was used to observe and analyze the differentially expressed proteins among three groups. **b** The heatmap illustrated 32 proteins with significant differences among three groups

Targeted nano-LC–MS/MS analysis showed that 4132 proteins were retained after preprocessing the original data from the myocardial tissue. Comparisons between the experimental and control groups revealed that the HFpEF group presented with 120 differentially expressed proteins, of which 54 were upregulated and 66 were downregulated. However, the intervention in the Cana group resulted in 13 of the HFpEF upregulated proteins being downregulated, and 13 of the downregulated proteins being upregulated. We then used a column diagram to identify and analyze these differentially expressed proteins (Fig. 4a) and found that these differential proteins could be displayed using hierarchical clustering analysis in the form of heat maps (Fig. 4b).

**Fig. 5:**
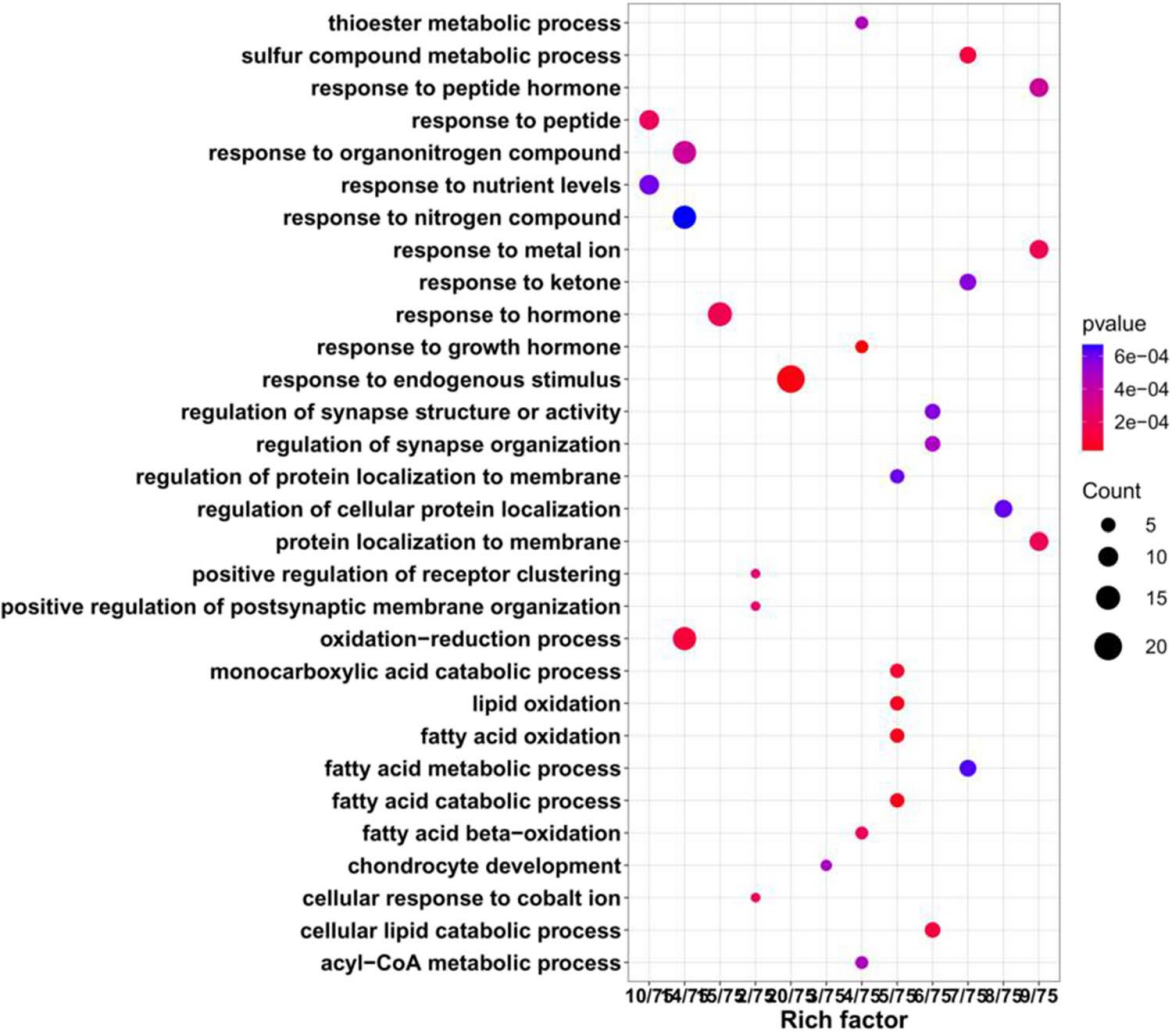
Enrichment of GO_BP analysis. The top 30 significantly enriched BP terms were presented. The *y*-axis showed the categories of GO terms and *x*-axis showed the rich factors

**Fig. 6:**
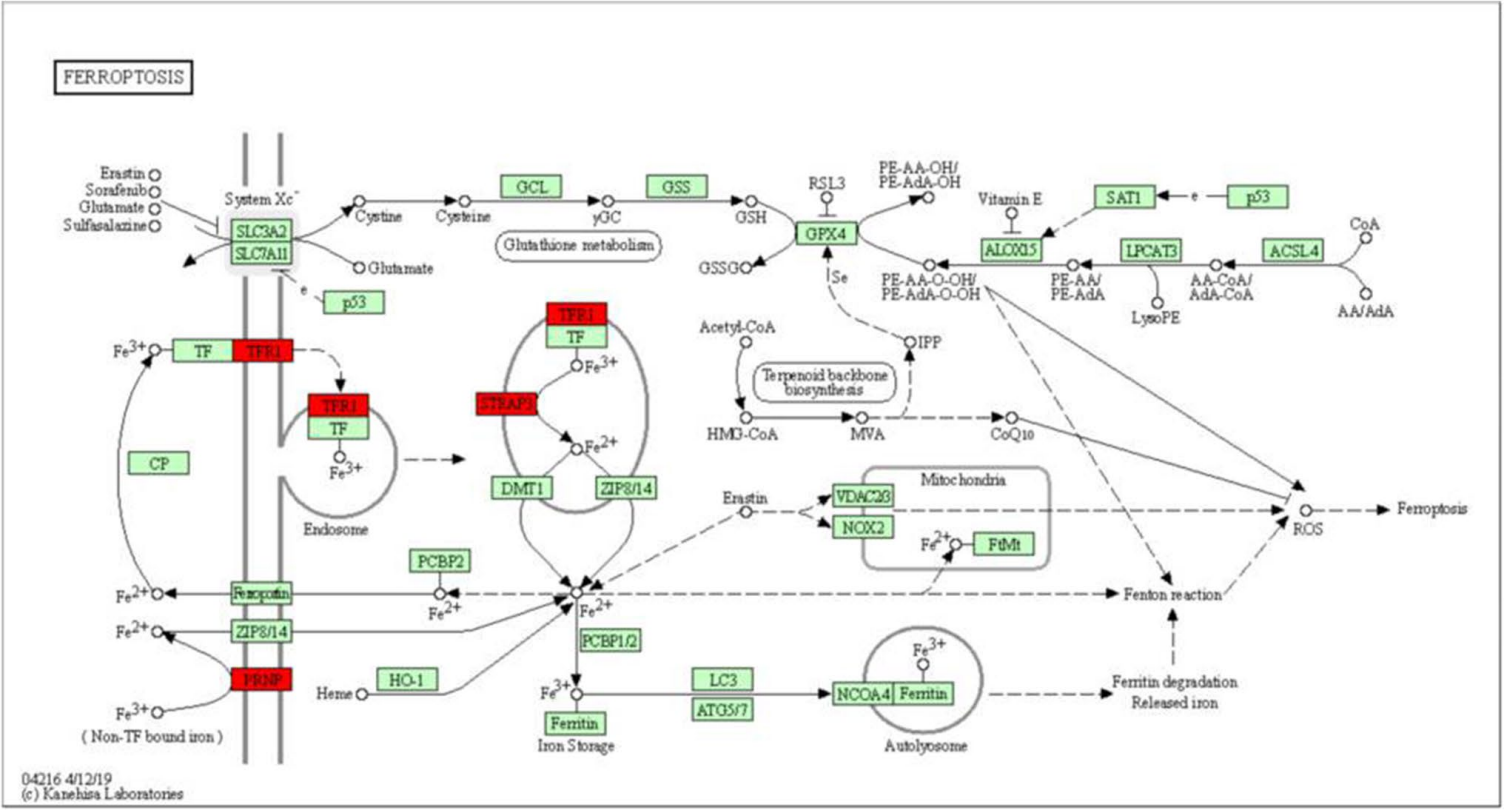
Ferroptosis pathway. The pathophysiological process of ferroptosis including iron metabolism, lipid peroxidation, amino acid metabolism, mevalonate pathway, and iron autophagy, etc. The red squares represented proteins that differ significantly between HFpEF group and Cana group. The green squares represented ferroptosis pathway-related proteins

A total of 84 of the differentially regulated proteins in the HFpEF and Cana group (Fig. 4a) were categorized using GO annotation, with the top 30 GO_BP enrichment results being summarized in Fig. 5. The significantly enriched metabolic pathways and their related proteins are shown in Table 4 and were identified using KEGG analysis. These differentially expressed proteins were concentrated in rno04216 Ferroptosis, rno03320 PPAR signaling pathway, rno04141 protein processing in endoplasmic reticulum, rno04310 Wnt signaling pathway, rno00190 oxidative phosphorylation, rno04146 peroxisome, rno04260 Cardiac muscle contraction, rno04714 Thermogenesis, and rno04723 retrograde endocannabinoid signaling. The enriched BP terms, including lipid oxidation, oxidation–reduction process, cellular lipid catabolic process, sulfur compound metabolic process, response to metal ion, protein localization to membrane, positive regulation of receptor clustering, regulation of protein localization to membrane, and regulation of cellular protein localization, were mainly associated with lipid metabolism, ion metabolism, sulfur compound metabolism, and the regulation of cellular protein localization, all of which were integrated within the ferroptosis metabolic pathway. Therefore, ferroptosis (Fig. 6) might be one of the key metabolic pathways responsible for the ameliorated HFpEF observed in this study.

**Table 4:** Enriched metabolic pathways and related proteins

KEGG pathway	Related proteins	*p* value
Ferroptosis	Tfrc, Steap3, Prnp	0.001492
PPAR signaling pathway	Acox1, Acaa1a, Ehhadh, Plin2, Hmgcs2	0.000096
Protein processing in endoplasmic reticulum	Pdia3, Dnajc10, Stt3b, Dnajb11	0.013082
Wnt signaling pathway	Serpinf1, Cacybp, Plcb3, Gpc4	0.010692
Oxidative phosphorylation	COX2, Cox5b, Cox6a1, Ndufb4	0.006973
Peroxisome	Acox1, Acaa1a, Ehhadh	0.012290
Cardiac muscle contraction	COX2, Cox5b, Cox6a1	0.011574
Thermogenesis	COX2, Cox5b, Cox6a1, Ndufb4	0.041364
Retrograde endocannabinoid signaling	Plcb3, Ndufb4, Gng10	0.049482

Related proteins were obtained from differentially expressed proteins between the HFpEF group and the Cana group

### Canagliflozin inhibited ferroptosis in HFpEF rats

**Fig. 7:**
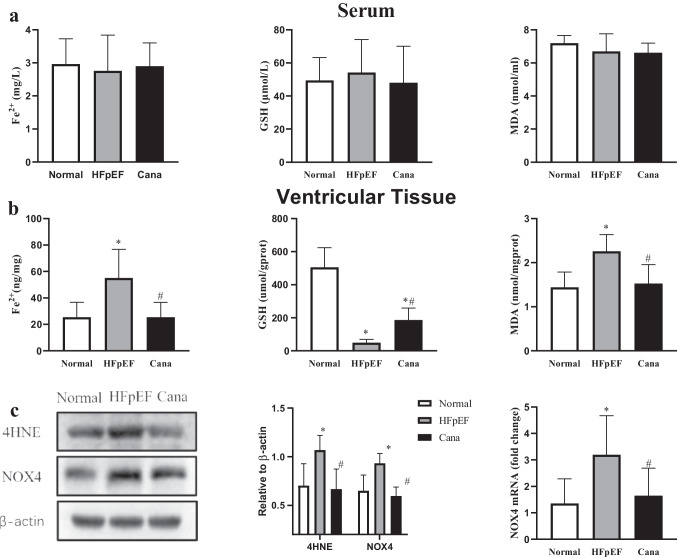
Effect of canagliflozin on ferroptosis in serum and cardiac tissue about lipid peroxidation levels. **a**, **b** The concentrations of Fe^2+^, GSH, and MDA in serum and ventricular tissue. **c** Western blotting analysis of 4HNE and NOX4 and PCR analysis of NOX4 in the rat myocardium. *n* = 6 rats/group. Data are presented as mean ± standard deviation. *p < 0.05 versus normal group. #*p* < 0.05 versus HFpEF group

**Fig. 8:**
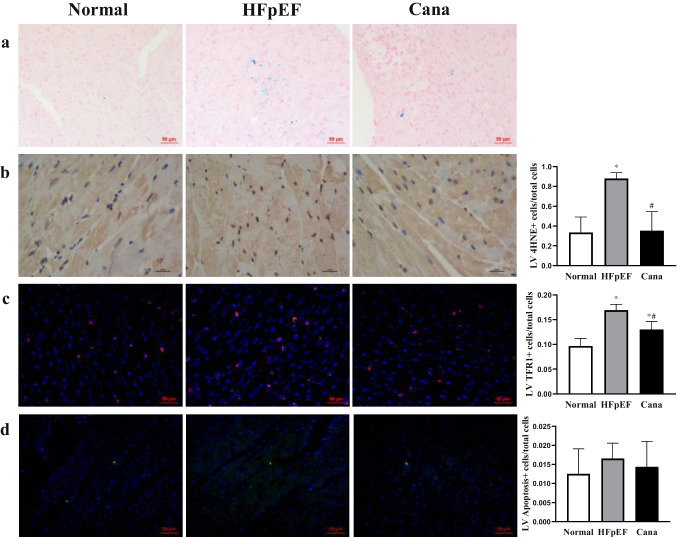
Effect of canagliflozin on ferroptosis and TUNEL analysis. **a** Representative images of Prussian blue iron staining for HFpEF-treated rat hearts. **b**, **c** Immunostaining of 4HNE and TFR1 protein expression in myocardium, followed by the percentages of positive cells in the total cells. **d** Dead cardiomyocytes measured by TUNEL staining. *n* = 6 rats/group. Data are presented as mean ± standard deviation. *p < 0.05 versus normal group. #*p* < 0.05 versus HFpEF group

**Fig. 9:**
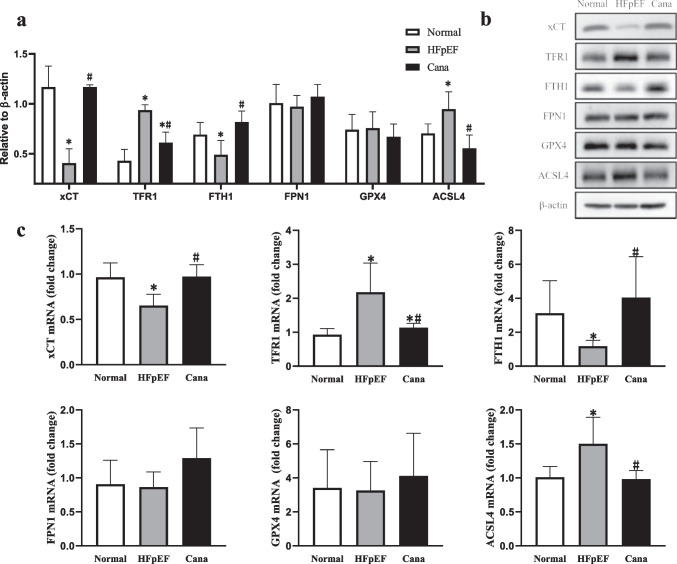
Effect of canagliflozin on ferroptosis about iron metabolism. **a**, **b** Western blotting analysis of xCT, TFR1, FTH1, FPN1, GPX4, and ACSL4. **c** PCR analysis of xCT, TFR1, FTH1, FPN1, GPX4, and ACSL4. *n* = 6 rats/group. Data are presented as mean ± standard deviation. *p < 0.05 versus normal group. #*p* < 0.05 versus HFpEF group

Given this data, we went on to evaluate the concentrations of Fe^2+^ (Figs. 7a and b, 8a), GSH (Fig. 7a, b) and MDA (Fig. 7a, b), and the expression of 4HNE (Figs. 7c and 8b) and NOX4 (Fig. 7c) in serum and ventricular tissues. We also examined the expression of xCT (Fig. 9), iron uptake protein TFR1 (Figs. 8c and 9), iron storage protein FTH1 (Fig. 9), iron release protein FPN1 (Fig. 9), key enzyme ACSL4 (Fig. 9), and GPX4 (Fig. 9), which are all key factors in ferroptosis. Our results showed that the concentrations of Fe^2+^, MDA, and the expression of TFR1, ACSL4, 4HNE, and NOX4 were all obviously increased in HFpEF when compared to the control, while all these indexes were significantly reduced in the Cana rats compared to the HFpEF rats. At the same time, the concentrations of GSH and the expression of xCT and FTH1 were significantly lower in HFpEF than in Normal rats, while these indexes were increased in the Cana rats when compared to the HFpEF rats. However, the protein expression of GPX4 and FPN1 showed little difference in the myocardial tissues of these three groups. The serum concentrations of Fe^2+^, MDA, and GSH were also evaluated, but there were no significant differences in these values between the three treatment groups. The results demonstrate that ferroptosis did exist in the development of HFpEF and canagliflozin could regulate ferroptosis by reducing the iron load and the accumulation of lipid peroxidation during HFpEF, while no more apoptotic cells were found in TUNEL staining in HFpEF rats compared with those in normal and Cana rats (Fig. 8d).

## Discussion

SGLT2i is a newly developed drug used in the treatment of type 2 diabetes mellitus independent from insulin. In addition to its excellent glycaemic effect, there is much evidence of the extra-glycemic benefits of SGLT2i, such as weight loss; decrease in blood pressure, uric acid levels, and TGs; increase in haematocrit and blood viscosity; and slowdown of kidney and heart disease progression (Irace et al. 2018; Lahnwong et al. 2018; Ndibalema et al. 2020). However, the significant reductions in the incidence of major adverse cardiovascular events could not be attributed solely to their glucose-lowering effects since studies have shown that, in nondiabetic models with cardiovascular dysfunction, SGLT2i could also show their benefits without hypoglycemic effect. They can prevent LV hypertrophy and fibrosis (Takasu and Takakura 2019), improve hemodynamics (Lee et al. 2019), reduce ferroptosis, apoptosis and inflammation through NLRP3 and MyD88-related pathway (Quagliariello et al. 2021), restrain sympathetic tone in the aorta, leading to NO-cGMP-PKG pathway activation (Zhang et al. 2019), lower infarct size, and ameliorate mitochondrial functions by reducing mitochondrial fission (Tanajak et al. 2018), as well as improving myocardial energetics by switching energy utilization from glucose to fatty acids, ketone bodies, and branched-chain amino acid (Santos-Gallego et al. 2019). But, there is no consensus on the exact underlying mechanism and overall effects of these treatments.

In clinical, the syndrome of HFpEF is heterogeneous and associated with other comorbidities, which probably lead to most clinical trials on treatments for HFpEF with neutral results. But, SGLT2i raised new hope. Given the fact that the underlying mechanisms supporting the potential cardiovascular benefits of SGLT2i remain unknown, especially for HFpEF, we designed this study using the DSS rat-induced HFpEF model with canagliflozin intervention. High-salt diet could cause renal dysfunction which contribute to the development of hypertension and lead to HFpEF in DSS rats. After 12 weeks of a high-salt diet, the cardiac diastolic dysfunction come up (Kim-Mitsuyama et al. 2004), and at 16–20 weeks of a high-salt diet, cardiac enlargement and a decrease in LVEF gradually occur. So HFpEF might shift to HFrEF in the later stage. This rat model is suitable for the study of molecular pathways and mechanisms in HFpEF, although it only represents patients suffering from salt-sensitive hypertension (Klotz et al. 2006). In our HFpEF group, we saw increased serum creatinine and blood pressure, giving rise to the formation of HFpEF with impaired CO and prolonged IVRT. And, canagliflozin could lower rats, body weight with increased urine output; decrease SBP, DBP, and MAP; promote ketone body formation; improve cardiac remodeling and function; and ameliorate renal function. But, it did not affect the level of serum lipid, glucose, and insulin. So to nondiabetic rats, canagliflozin did not cause hypoglycemia. As we saw ketone effects from canagliflozin, another study in our team investigated myocardial metabolism and found canagliflozin reduced myocardial glucose metabolism and increased fatty acid metabolism and ketogenesis in HFpEF rats possibly through the activation of AMPK/SIRT1/PGC-1a pathway. Clinical trials have been failing to identify effective treatments for HFpEF but evidence supports the use of diuretics, mineralocorticoid antagonists, and lifestyle interventions (Borlaug 2020). We know SGLT2i has osmotic diuretic and mild natriuretic effects and could reduce body weight which contribute to the clinical benefits even though the pathophysiological mechanism is complex and not fully understood. Besides, antihypertensive treatment is also important for the control of HFpEF and SGLT2i could decrease BP through diuretic effect, reducing the motor activity of pulse stiffness, improving renal function and endothelial function, etc. (Chilton et al. 2015). Another group in our research team conducted relevant studies. They also used DSS rats to construct HFpEF models and divided the rats into five groups: control group, HFpEF group, irbesartan intervention group, canagliflozin intervention group, and irbesartan combined with the canagliflozin intervention group. The results showed that although the decrease extent of BP in the irbesartan intervention group was better than that in the canagliflozin intervention group, there was no statistical difference between the two groups, and the decrease of BP in the combined treatment group was more obvious, almost close to the control group. That was, in terms of BP, the antihypertensive effect between canagliflozin and irbesartan was equivalent. However, Krasnova found that, in the male Wistar rats model with chronic HF induced by myocardial infarction, the left ventricular volume, EF, and the other echocardiographic data were not changed significantly after treatment with empagliflozin for 3 months. And, the exercise tolerance in these rats increased markedly. While treatment with fosinopril aroused that the left atrial anterior–posterior dimension and right atrium long axis dimension increased, the left ventricular volume and EF were unchanged. But, the exercise tolerance was observably decreased. Therefore, it was concluded that compared with fosinopril, empagliflozin reduced the progression of left ventricular dysfunction and improved tolerance to physical exercise in rats with HF (Krasnova et al. 2020). Thus, for normoglycemic and non-hypertensive rats, empagliflozin could get more cardiac benefits than antihypertensive when it did not need blood pressure therapy or hypoglycemic agent. Therefore, we deduced that lowering BP simply might not be enough for the improvement of HFpEF, and the benefits of canagliflozin on HFpEF might be due to its direct effect on the heart. For further research, proteomic was carried out to help us to identify the likely regulatory or metabolic mechanism affected by canagliflozin. With significantly different proteins between HFpEF rats and Cana rats, KEGG analysis showed ferroptosis was a remarkable metabolic pathway, and GO_BP enrichment results then revealed that 9 of the top 30 significantly enriched GO terms were associated with ferroptosis. Iron is an essential micronutrient required for mitochondrial function, DNA synthesis, and other cellular processes. Both iron overload and iron deficiency are interconnected with cardiomyopathy and heart failure (Gulati et al. 2014; Lakhal-Littleton et al. 2015; Xu et al. 2015). These findings underscore the importance of maintaining iron homeostasis within the heart. Ferroptosis, an iron-dependent form of regulated necrosis, is a novel mechanism for programmed cell death closely associated with iron homeostasis, lipid peroxidation, and oxidative stress. Existing studies have reported that ferroptosis participates in the pathological process of cardiovascular diseases. For myocardial ischemia/reperfusion injury and cardiomyopathy it induced, the mTOR pathway may protect cardiomyocytes against ferroptosis because of the reduction of ROS production (Baba et al. 2018), and Icariin attenuates ferroptosis by activating the Nrf2/HO-1 signaling pathway (Liu et al. 2021). Doxorubicin can upregulate heme oxygenase-1 to release free iron in cardiomyocyte, resulting in ferroptotic cardiomyopathy with HF (Fang et al. 2019). On the other side, deferoxamine and dexrazoxane are iron chelators which can alleviate myocardial infarction or HF induced by ischemia/reperfusion (Gao et al. 2015). Lapenna et al. (2018) showed that the oxidation levels of low oxygen molecular weight iron, lipids, and proteins in the heart of old rabbits were generally increased when compared to younger rabbits. Therefore, ferroptosis was likely to be a potential mechanism of cell death during cardiac dysfunction. In addition, Liu et al. (2018) indicated that puerarin played a role in inhibiting cardiomyocyte death by restricting ferroptosis during HF both in vivo and in vitro. Wang et al. (2020a, b) suggested that in the late stages of HF, myocardial fibrosis may be caused by ferroptosis regulated by JNK/p53 signaling mediated changes in MLK3. In isoproterenol-induced HF mice, atorvastatin suppressed ferroptosis through inhibiting ferritinophagy (Ning et al. 2021). Taken together, these studies suggest that ferroptosis may play a key role in the development of myocardial ischemia/reperfusion injury, cardiomyopathy, and HF. But, rare relevant studies have confirmed the role of ferroptosis in a HFpEF model with SGLT2i intervention. Since inhibition of ferroptosis can suppress oxidative stress, which is considered to be the common basis of diabetes and cardiovascular diseases, that is the “common soil” hypothesis (Pradeepa et al. 2012), we believe that this may be the key point to improve the insulin resistance in diabetes mellitus and the prognosis of cardiovascular diseases. Therefore, we went on to perform several relevant verifications.

First, the pathophysiology suggests that (Fig. 6) Fe^3+^ may enter the inclusion bodies via TFR1 and is then reduced to Fe^2+^ by the six-transmembrane epithelial antigen of prostate 3. This Fe^2+^ then plays a critical physiological role in the cytoplasmic labile iron pool after being released by divalent metal ion transporters or zinc and iron transporters. Redundant iron is then stored in the FTL and FTH1 in the form of Fe^3+^ or transported outside the cell using iron transporter1 (FPN1) (Torti and Torti 2013). Our results showed HFpEF rats experienced a significant increase in TFR1 expression, decrease in FTH1 expression, and stable FPN1 protein expression when compared to the control, which inferred that the free iron and iron loading in the unstable iron pool were increased. This inference was further confirmed by Prussian blue iron staining and biochemical testing and the data showed the concentration of Fe2 + in HFpEF rats was significantly higher than that of the Normal rats. However, the indicators above were reversed by canaliflozin with iron overload improved supporting its likely interaction with ferroptosis. In addition, cells experiencing iron overload may undergo Fenton reactions with hydrogen peroxide to form hydroxyl radicals and ROS, which then damage nucleic acids, proteins, and cell membranes (Li et al. 2012). Polyunsaturated fatty acids in the phospholipids of the cell and organelle membranes are dominated by arachidonic and adrenic acids, which are acylated by ACSL4, and esterified by lysophosphatidylcholine acyltransferase 3 to produce polyunsaturated fatty acid phospholipids. Polyunsaturated fatty acid phospholipids are less stable in structure and more easily oxidized, which may result in the increased production of lipid peroxides by the lipoxygenases, with the major products of these reactions being 4-HNE and MDA (Feng and Stockwell 2018), and where ACSL4 acts as the key to adjusting the lipid metabolism pathway (Doll et al. 2017). MDA is also used as a marker of oxidative stress. Under the catalysis of GPX4, known as a marker of ferroptosis, GSH can convert lipid peroxides into non-toxic fatty alcohols, suggesting that GSH has strong reducing properties and plays an important role in the antioxidant mechanism of cells (Friedmann Angeli et al. 2014). Moreover, reduced nicotinamide adenine dinucleotide phosphate oxidase had been shown to act as the primary oxidase in the cardiovascular system and one of the main sources of ROS. Of these proteins, NOX4 is known to make the most significant contribution to myocardial hypertrophy and is regarded as a cardiac reflection of oxidative stress (Schnelle et al. 2021). Therefore, we estimated the level of lipid peroxidation and oxidative stress by evaluating the expression levels of ACSL4, GPX4, 4-HNE, and NOX4 in myocardial tissue, as well as MDA and GSH content. The results revealed that HFpEF induced lipid peroxidation and oxidative stress accompanied by a significant increase in ACSL4, 4-HNE and NOX4 expression, MDA content, and decrease in GSH content, which may cause Fenton reactions and ferroptosis. Moreover, the indicators above were reversed by canagliflozin. But, the expression of GPX4 was not changed through the study. Researches have shown that there are two classes of ferroptosis inducers (Dixon et al. 2012). Class one, including erastin and sulfasalazine, causes ferroptosis by system Xc- inhibition that reduces GSH content. Class 2, including Ras-selective lethal 3 and DPI7 (also known as ML162), directly inhibits GPX4 enzymatic activity without depleting intracellular GSH. Then, we infer that the reduction in GSH content and inhibition of system Xc- may be pivotal in HFpEF rats, rather than GPX4 inactivation. Then, we confirmed this hypothesis through detecting the expression of xCT. Results showed when HFpEF occurred, xCT was suppressed and canagliflozin could reverse this suppression. Based on the fact present, we conclude that ferroptosis may be one of the pathogenesis in HFpEF, and canagliflozin treatment may regulate ferroptosis to ameliorate HFpEF partly via reducing iron intake and iron content in unstable iron pool, reducing lipid peroxidation, increasing GSH production, and suppressing oxidative stress.

Although there is evidence supporting satisfactory protection of canagliflozin in HFpEF, the beneficial effects of canagliflozin treatment on the regulation of ferroptosis in failing heart remain unveiled. Firstly, we can see autophagosomes in the representative TEM images from HFpEF rats, which are obviously more than Cana rats, indicating that autophagy might be one of the mechanism for canagliflozin to regulate ferroptosis. Atorvastatin has been verified that it exhibited protective effect on failing myocardium though inhibiting ferritinophagy-mediated ferroptosis. But, further studies about how it regulates the FTH1 autophagic axis need to be investigated in depth (Ning et al. 2021). Beside regulated necrosis—ferroptosis and autophagy—apoptosis is the third major type of cell death when HF occurs. Our apoptosis assay showed there was no significant difference among groups, and further validation is needed. Secondly, reports showed previously that the mechanistic target of rapamycin (mTOR) regulated iron homeostasis by modulating TFR1 stability and altering cellular iron flux (Bayeva et al. 2012), and inhibition of mTOR was related to microcytic anemia (Przybylowski et al. 2013), suggesting that mTOR played a crucial role in iron homeostasis, partly through TFR1. Baba et al. (2018) showed that mTOR played an important role in protecting cardiomyocytes against ferroptosis, at least in part by regulating ROS production. What’s more, both our proteomics and data highlighted the critical function of TFR1 in HFpEF and canagliflozin treatment, which indicated mTOR metabolic pathway might be essential in the process. Thirdly, aberrant NRF2 signaling leads to diseases connected with lipid peroxidation and ferroptosis (Dodson et al. 2019). And, the increasing expression of the target gene, HO-1, can degrade heme into ferrous iron and provide anti-apoptotic and antioxidant effects (Wang et al. 2020a, b). A report speculated that icariin reduced ferroptosis of cardiomyocytes by affecting the NRf2/HO-1 pathway. So the expressions of NRf2 and HO-1 were also important notably. Then, the mechanisms of ferroptosis are delicate and complex. In addition to the abovementioned abnormalities in the iron metabolism and lipid peroxidation pathways, the amino acid metabolism, mevalonate pathway, iron autophagy, and voltage-dependent anion channels can also all regulate ferroptosis (Dixon et al. 2012), which are not included in this study. Therefore, further evaluations of the fine molecular mechanism underlying canagliflozin-mediated regulation of ferroptosis and its influence on related proteins in the upstream pathways are required. Then, knockdown or overexpression of the related proteins needs to do to further study the mechanism of canagliflozin. What is more, ferroptosis can be inhibited by ferrostatins, liproxstatins, etc. Then, we should confirm that if the regulation of ferroptosis by SGLT2i is blocked in terms of cells, these cardiovascular benefits will exist or not. Our team is working on it.

## Conclusions

In summary, canagliflozin therapy improved high blood pressure and alleviated cardiac remodeling and left ventricular diastolic dysfunction in a rodent model of HFpEF. Potential pathophysiological mechanisms that underly these salutary changes are likely multifactorial. However, the regulated ferroptosis mechanism may play a crucial role in the process. Therefore, canagliflozin is a promising agent in the prevention and treatment of HFpEF. And, more clinical data are needed to verify the safety and efficacy of canagliflozin, and its potential mechanism also needs further exploration.

### Supplementary Information

Below is the link to the electronic supplementary material.

Supplementary file1 (XLS 71 KB)

Supplementary file2 (XLS 7303 KB)

Supplementary file3 (DOC 691 KB)

Supplementary file4 (DOC 17878 KB)

Supplementary file5 (XLSX 9 KB)

## Data Availability

Data for this research work were made available at submission.
