# Supplementary material for: Canagliflozin mitigates ferroptosis and ameliorates heart failure in rats with preserved ejection fraction
Source: Naunyn Schmiedebergs Arch Pharmacol. 2022 Apr 27;395(8):945–62. doi: 10.1007/s00210-022-02243-1 (PMC9276585; doi:10.1007/s00210-022-02243-1)
Supplement: Supplementary file 3 — Supplementary file3 (DOC 691 KB) [file 210_2022_2243_MOESM3_ESM.doc]

Western blot

4HNE
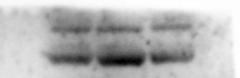
 4HNE
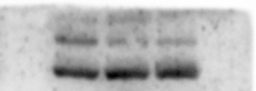


β-actin
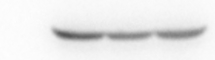
 β-actin
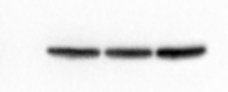


Normal HFpEF Cana Normal HFpEF Cana

4HNE
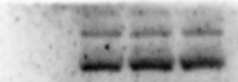
 4HNE
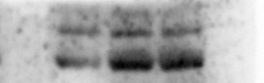


β-actin
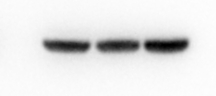
 β-actin
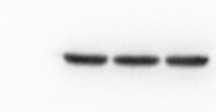


Normal HFpEF Cana Normal HFpEF Cana

4HNE
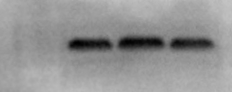
 4HNE
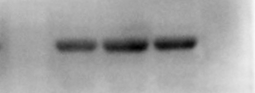


β-actin
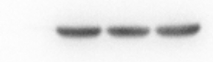
 β-actin
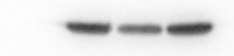


Normal HFpEF Cana Normal HFpEF Cana

NOX4
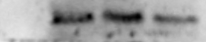
 NOX4
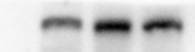


β-actin
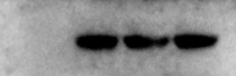
 β-actin
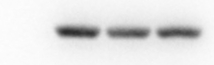


Normal HFpEF Cana Normal HFpEF Cana

NOX4
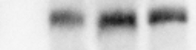
 NOX4
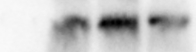


β-actin
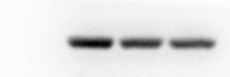
 β-actin
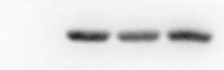


Normal HFpEF Cana Normal HFpEF Cana

NOX4
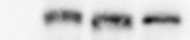
 NOX4
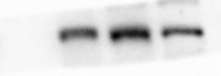


β-actin
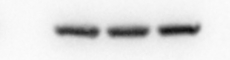
 β-actin
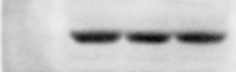


Normal HFpEF Cana Normal HFpEF Cana

|  | Normal | HFpEF | Cana |
| --- | --- | --- | --- |
| 4HNE | 0.366581578 | 1.096015001 | 0.307229187 |
| 4HNE | 0.587569686 | 0.840687031 | 0.557995289 |
| 4HNE | 0.830982827 | 1.085083272 | 0.705453652 |
| 4HNE | 0.897528296 | 1.014818909 | 0.73599635 |
| 4HNE | 0.584094476 | 1.060774787 | 0.824037907 |
| 4HNE | 0.946768802 | 1.307178262 | 0.865847101 |
| NOX4 | 0.846223064 | 0.955133429 | 0.424074078 |
| NOX4 | 0.364939562 | 0.852712085 | 0.713091995 |
| NOX4 | 0.526587975 | 1.013798411 | 0.639391024 |
| NOX4 | 0.605886642 | 0.873524853 | 0.545112554 |
| NOX4 | 0.75381913 | 1.022386294 | 0.638705646 |
| NOX4 | 0.761995611 | 1.033343025 | 0.584708911 |

**Fig.7.c representative images of Western blots and optical density for Western blots of 4HNE and NOX4 against β-actin.**

TFR1
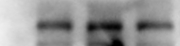
 TFR1
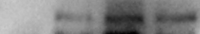


β-actin
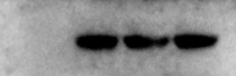
 β-actin
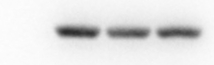


Normal HFpEF Cana Normal HFpEF Cana

TFR1
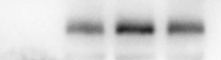
 TFR1
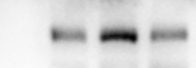


β-actin
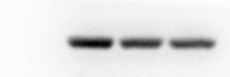
 β-actin
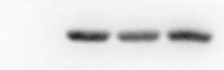


Normal HFpEF Cana Normal HFpEF Cana

TFR1
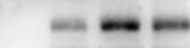
 TFR1
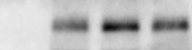


β-actin
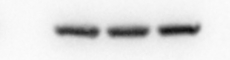
 β-actin
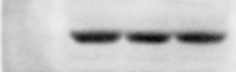


Normal HFpEF Cana Normal HFpEF Cana

FTH1
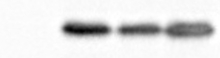
 FTH1
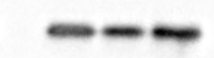


β-actin
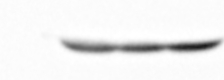
 β-actin
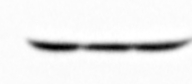


Normal HFpEF Cana Normal HFpEF Cana

FTH1
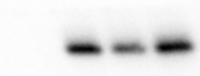
 FTH1
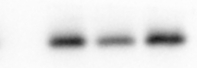


β-actin
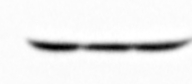
 β-actin
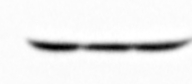


Normal HFpEF Cana Normal HFpEF Cana

FTH
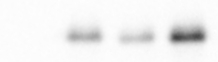
 FTH1
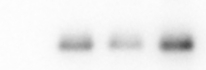


β-actin
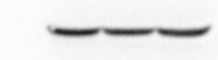
 β-actin
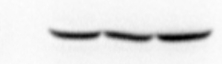


Normal HFpEF Cana Normal HFpEF Cana

FPN1
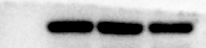
 FPN1
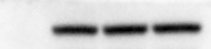


β-actin
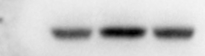
 β-actin
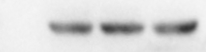


Normal HFpEF Cana Normal HFpEF Cana

FPN1
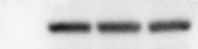
 FPN1
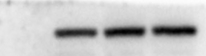


β-actin
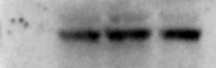
 β-actin
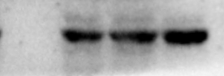


Normal HFpEF Cana Normal HFpEF Cana

FPN1
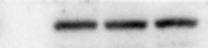
 FPN1
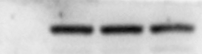


β-actin
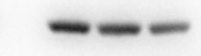
 β-actin
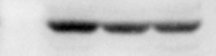


Normal HFpEF Cana Normal HFpEF Cana

GPX4
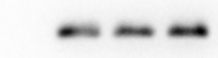
 GPX4
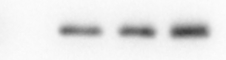


β-actin
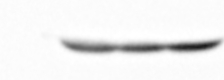
 β-actin
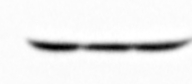


Normal HFpEF Cana Normal HFpEF Cana

GPX4
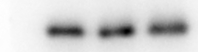
 GPX4
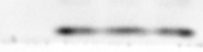


β-actin
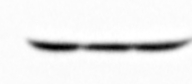
 β-actin
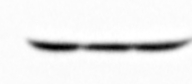


Normal HFpEF Cana Normal HFpEF Cana

GPX4
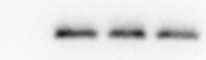
 GPX4
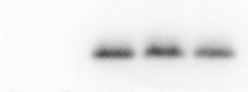


β-actin
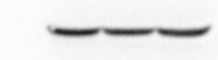
 β-actin
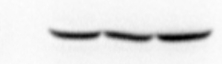


Normal HFpEF Cana Normal HFpEF Cana

ACSL4
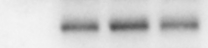
 ACSL
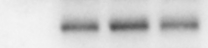


β-actin
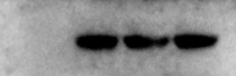
 β-acti
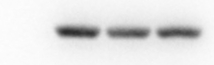


Normal HFpEF Cana Normal HFpEF Cana

ACSL4
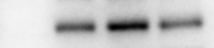
 ACSL4
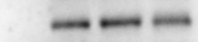


β-actin
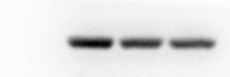
 β-actin
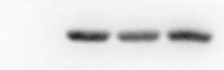


Normal HFpEF Cana Normal HFpEF Cana

ACSL4
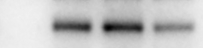
 ACSL4
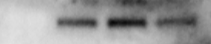


β-actin
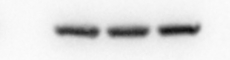
 β-actin
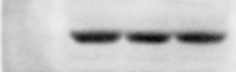


Normal HFpEF Cana Normal HFpEF Cana

|  | Normal | HFpEF | Cana |
| --- | --- | --- | --- |
| TFR1 | 0.600269284 | 1.019859182 | 0.647388394 |
| TFR1 | 0.389593349 | 0.887713181 | 0.689095878 |
| TFR1 | 0.498014968 | 0.962138222 | 0.467180568 |
| TFR1 | 0.260159087 | 0.892039588 | 0.724555766 |
| TFR1 | 0.501700079 | 0.899581825 | 0.702275451 |
| TFR1 | 0.326691531 | 0.902972758 | 0.546072412 |
| FTH1 | 0.787414076 | 0.685460394 | 0.83961552 |
| FTH1 | 0.776821705 | 0.659584991 | 0.942070306 |
| FTH1 | 0.581748064 | 0.374909423 | 0.642978626 |
| FTH1 | 0.852418681 | 0.588777343 | 0.871011595 |
| FTH1 | 0.475457814 | 0.296437051 | 0.810891537 |
| FTH1 | 0.634637867 | 0.366285289 | 0.718469801 |
| FPN1 | 1.232043675 | 0.832899988 | 0.993558152 |
| FPN1 | 1.14802773 | 1.056312129 | 1.108788059 |
| FPN1 | 1.186432305 | 0.914997365 | 1.095606013 |
| FPN1 | 0.848829288 | 0.906892722 | 0.96440477 |
| FPN1 | 0.939631648 | 0.916773973 | 1.230061927 |
| FPN1 | 0.723704906 | 0.999407593 | 0.906269411 |
| GPX4 | 0.571510582 | 0.615874103 | 0.673733673 |
| GPX4 | 0.502924667 | 0.573328874 | 0.736410014 |
| GPX4 | 0.65492412 | 0.658476842 | 0.603555382 |
| GPX4 | 0.774000501 | 0.632392293 | 0.634862139 |
| GPX4 | 0.84790407 | 0.782898938 | 0.661151858 |
| GPX4 | 0.868834144 | 0.806893637 | 0.494271873 |
| ACSL4 | 0.671974139 | 1.051553013 | 0.673883988 |
| ACSL4 | 0.800085784 | 1.007675843 | 0.495798582 |
| ACSL4 | 0.60270369 | 1.010917442 | 0.646658068 |
| ACSL4 | 0.829919518 | 1.17953089 | 0.385311592 |
| ACSL4 | 0.781486633 | 0.924449505 | 0.72597045 |
| ACSL4 | 0.732280807 | 1.016097697 | 0.642639769 |

**Fig.9.b representative images of Western blots and optical density for Western blots of TFR1, FTH1, FPN1, GPX4 and ACSL4 against β-actin.**

**xCT
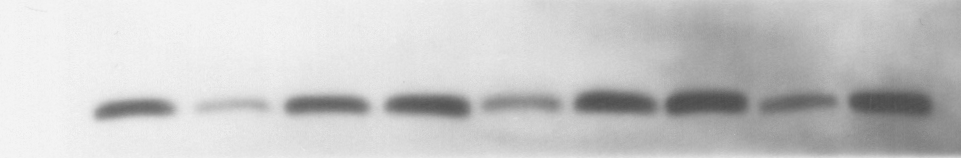
**

β-actin  **
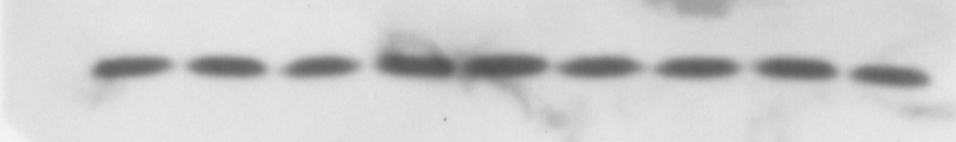
**

|  | Normal | HFpEF | Cana |
| --- | --- | --- | --- |
| **xCT** | 0.953023914 | 0.261757322 | 1.18258825 |
| **xCT** | 1.374212466 | 0.40318242 | 1.143650441 |
| **xCT** | 1.176496981 | 0.550660412 | 1.179108351 |

**Fig.9.b representative images of Western blots and optical density for Western blots of xCT against β-actin.**
