# Supplementary material for: Canagliflozin mitigates ferroptosis and ameliorates heart failure in rats with preserved ejection fraction
Source: Naunyn Schmiedebergs Arch Pharmacol. 2022 Apr 27;395(8):945–62. doi: 10.1007/s00210-022-02243-1 (PMC9276585; doi:10.1007/s00210-022-02243-1)

The original images presented in figure 2d (mid panel).


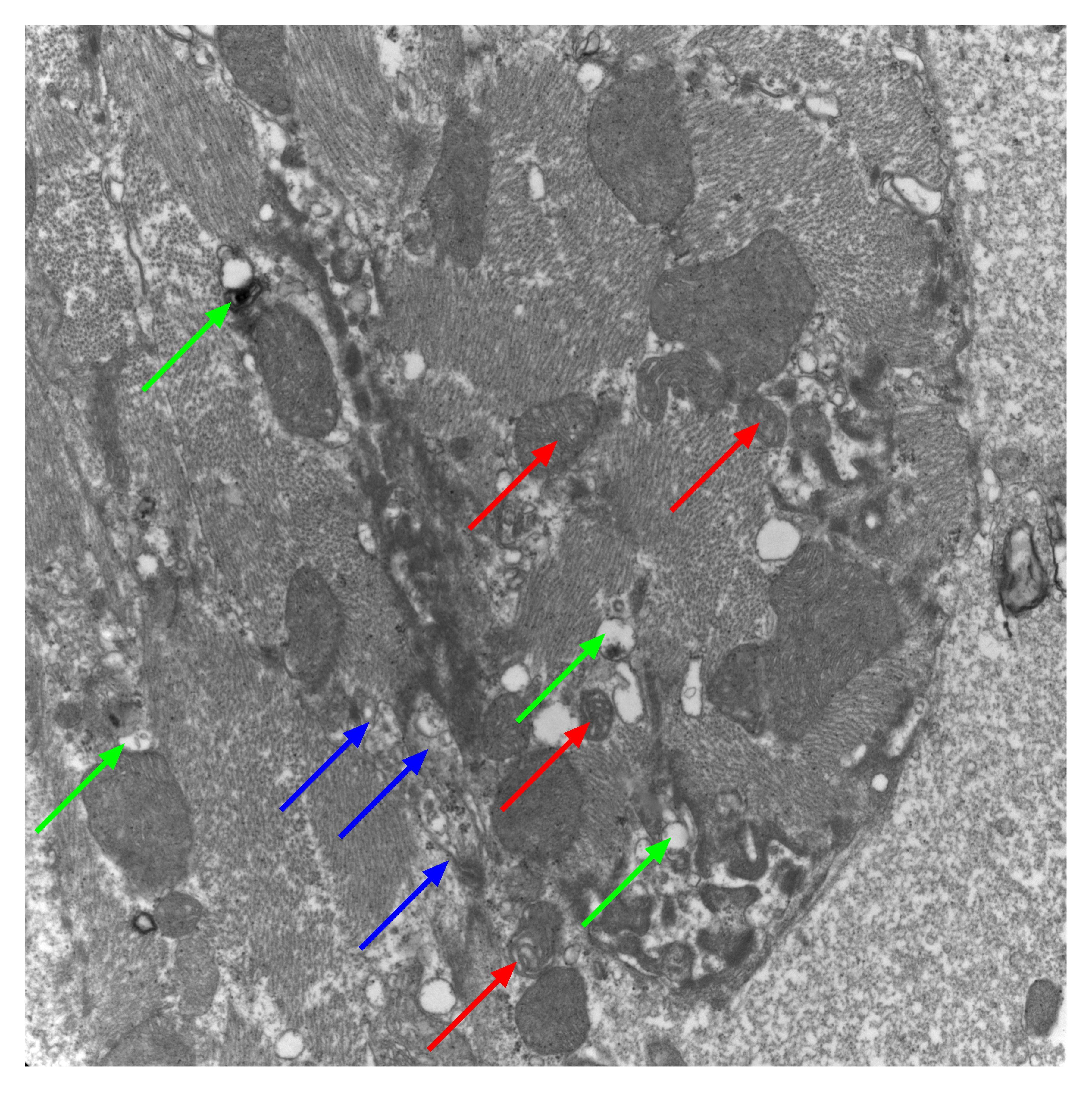


The original images presented in figure 8 with bar graph data.

Normal group HFpEF group Cana group


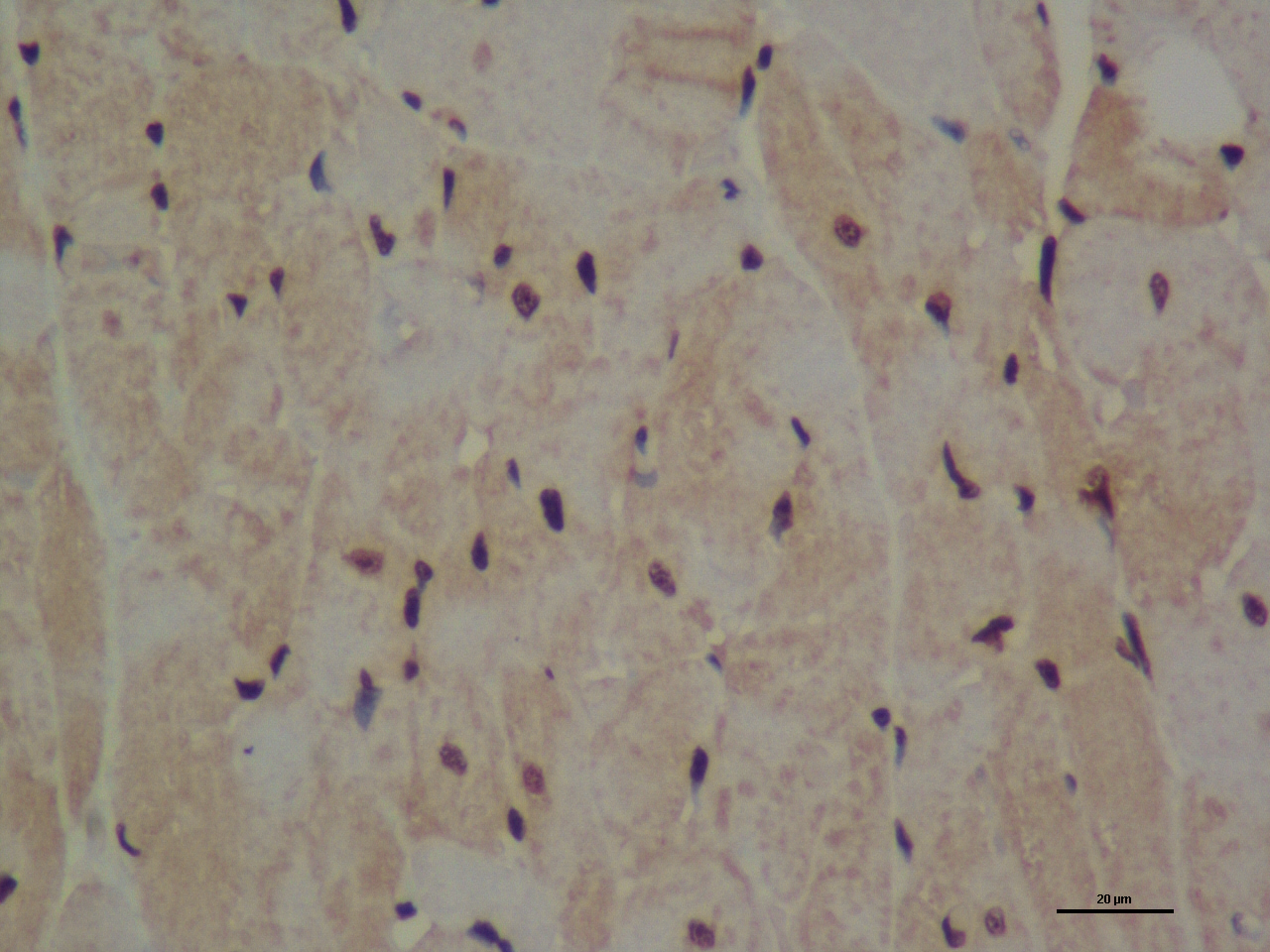

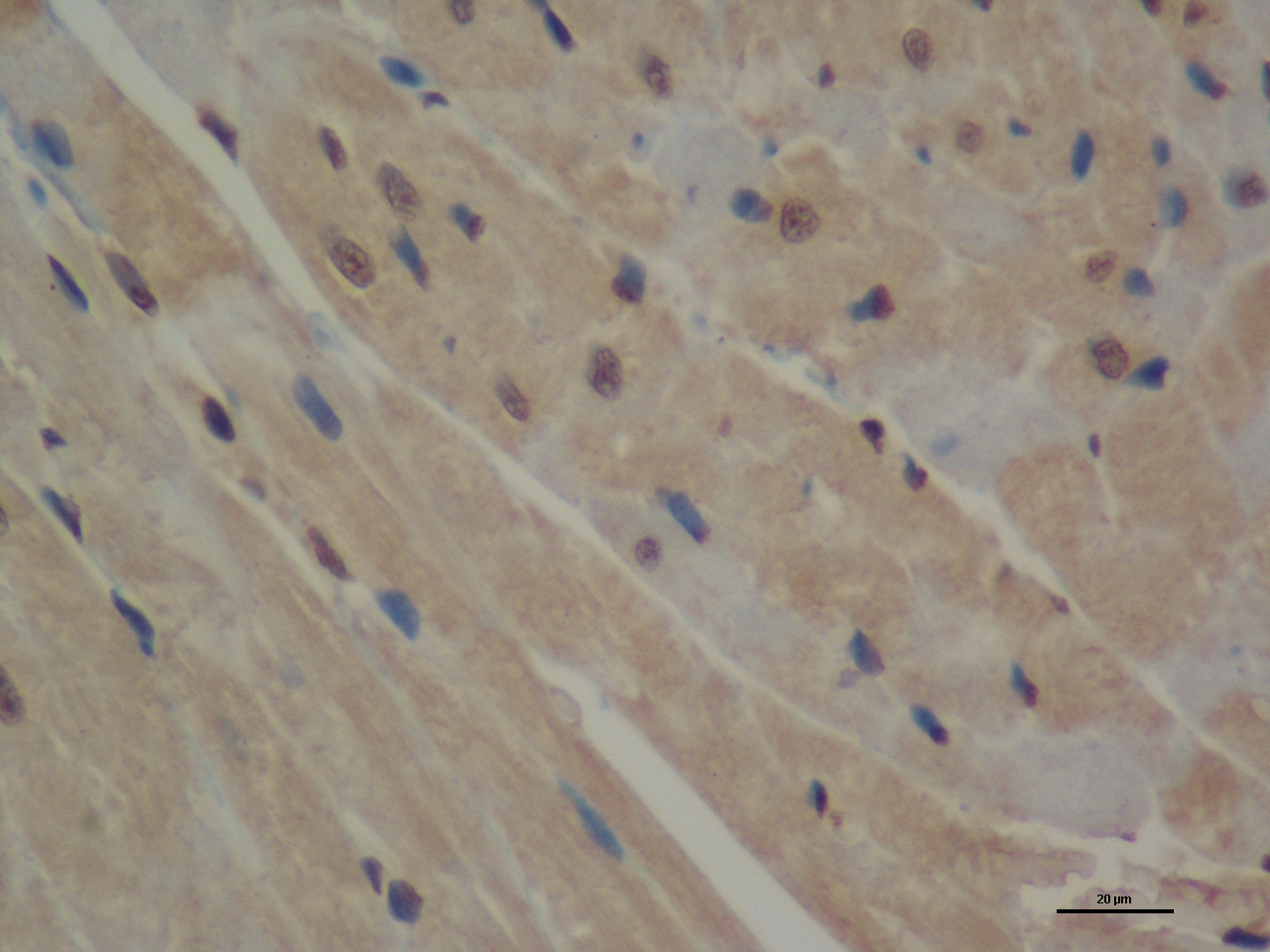

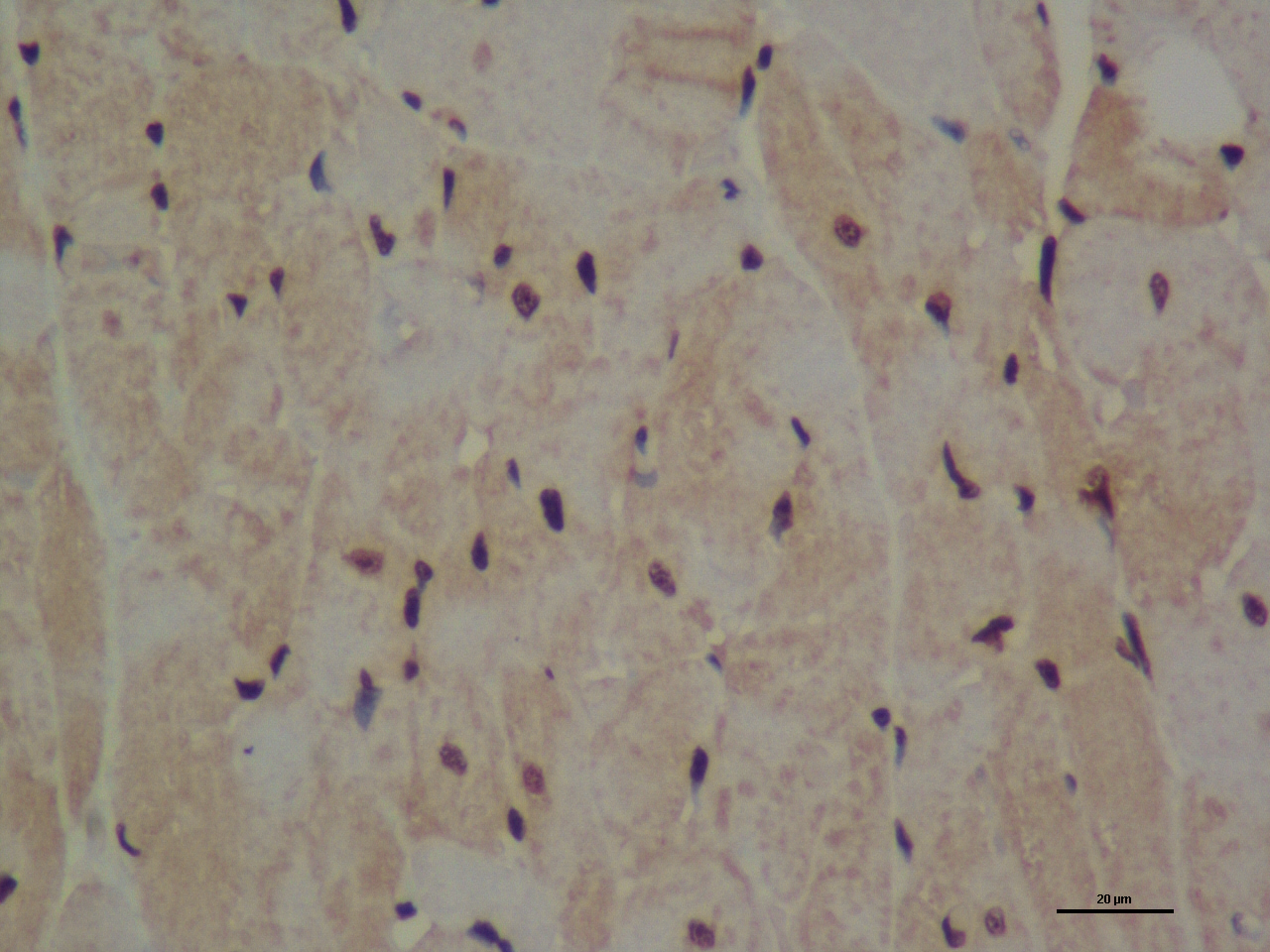

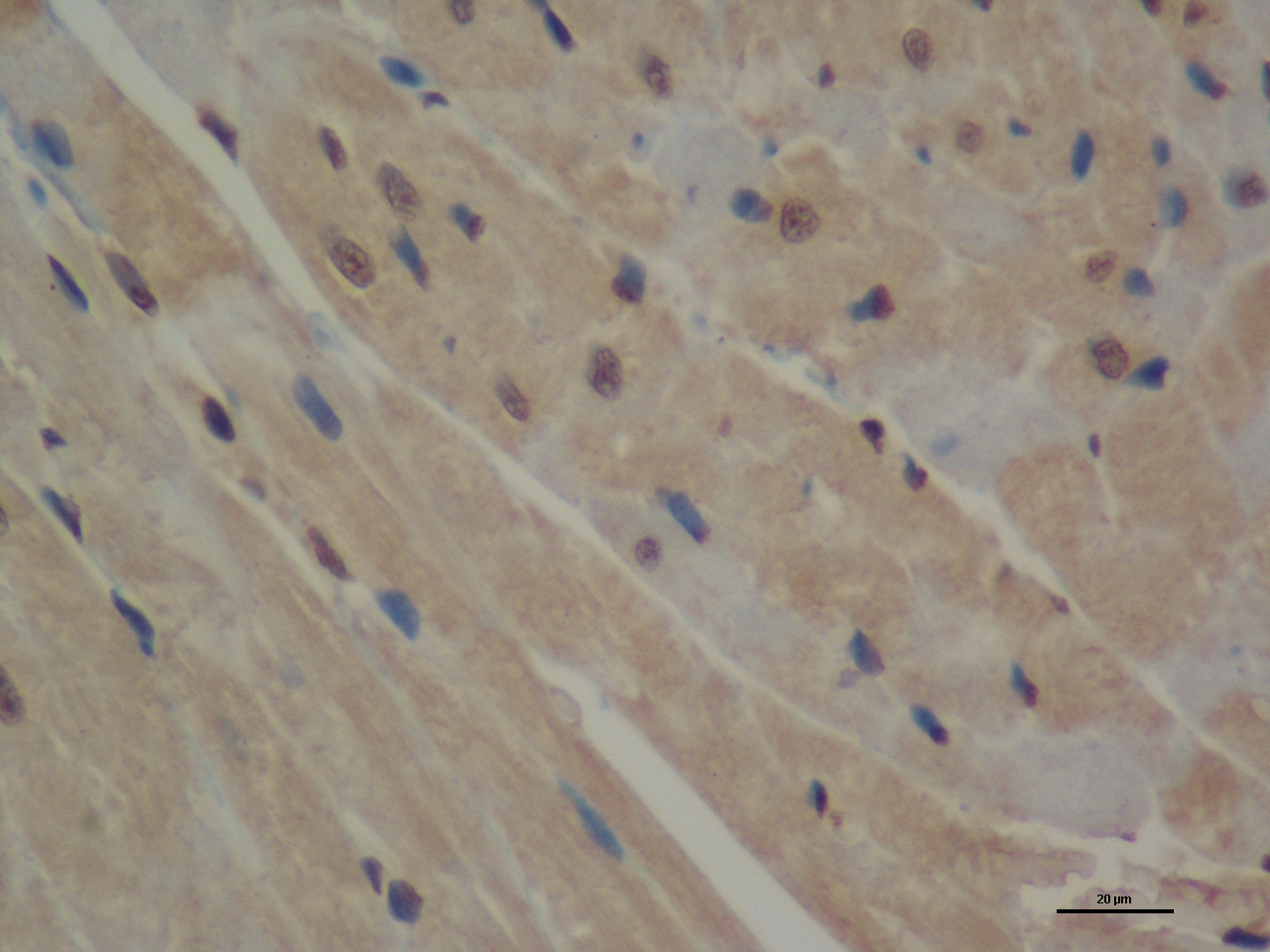

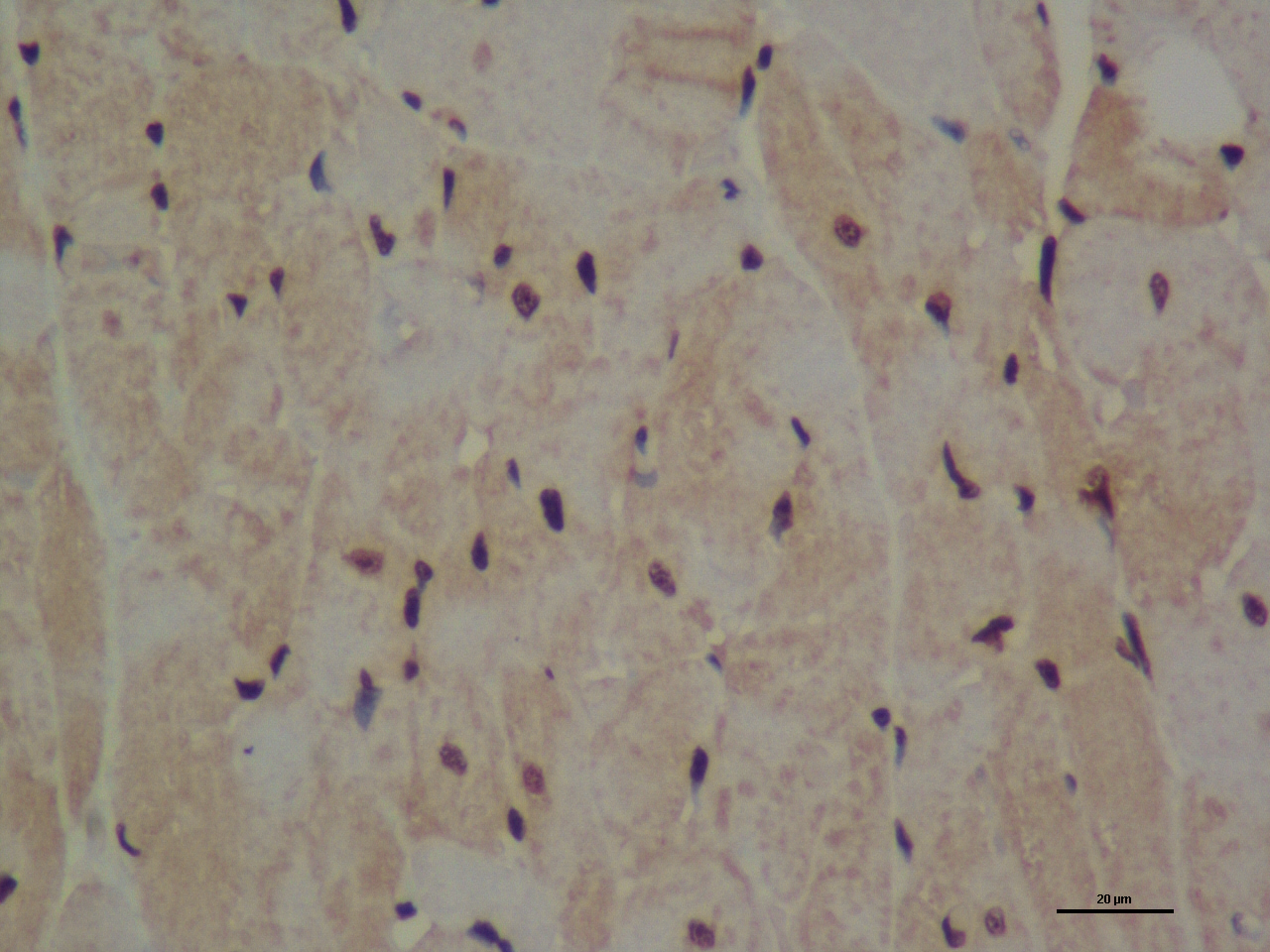

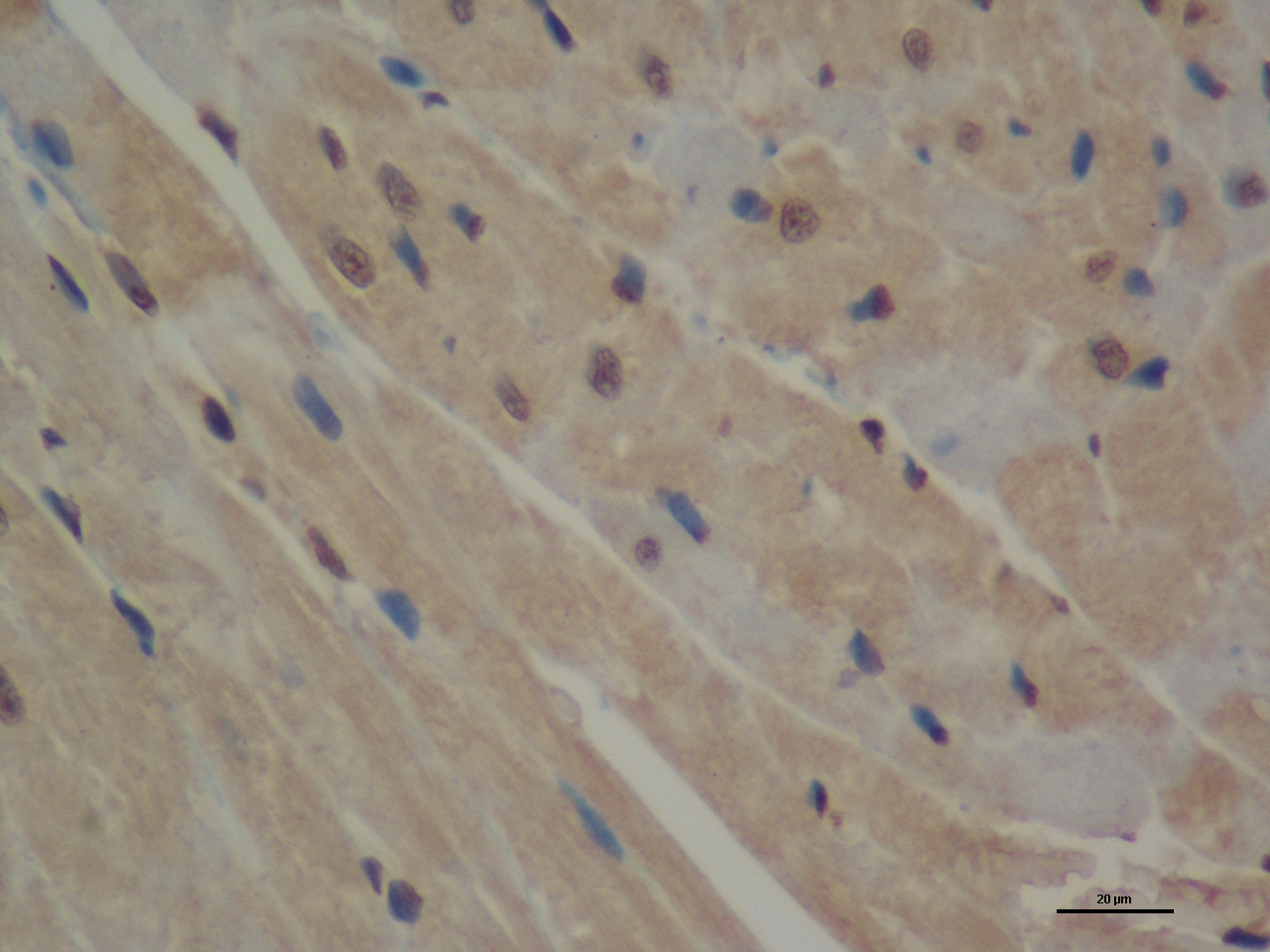
 c


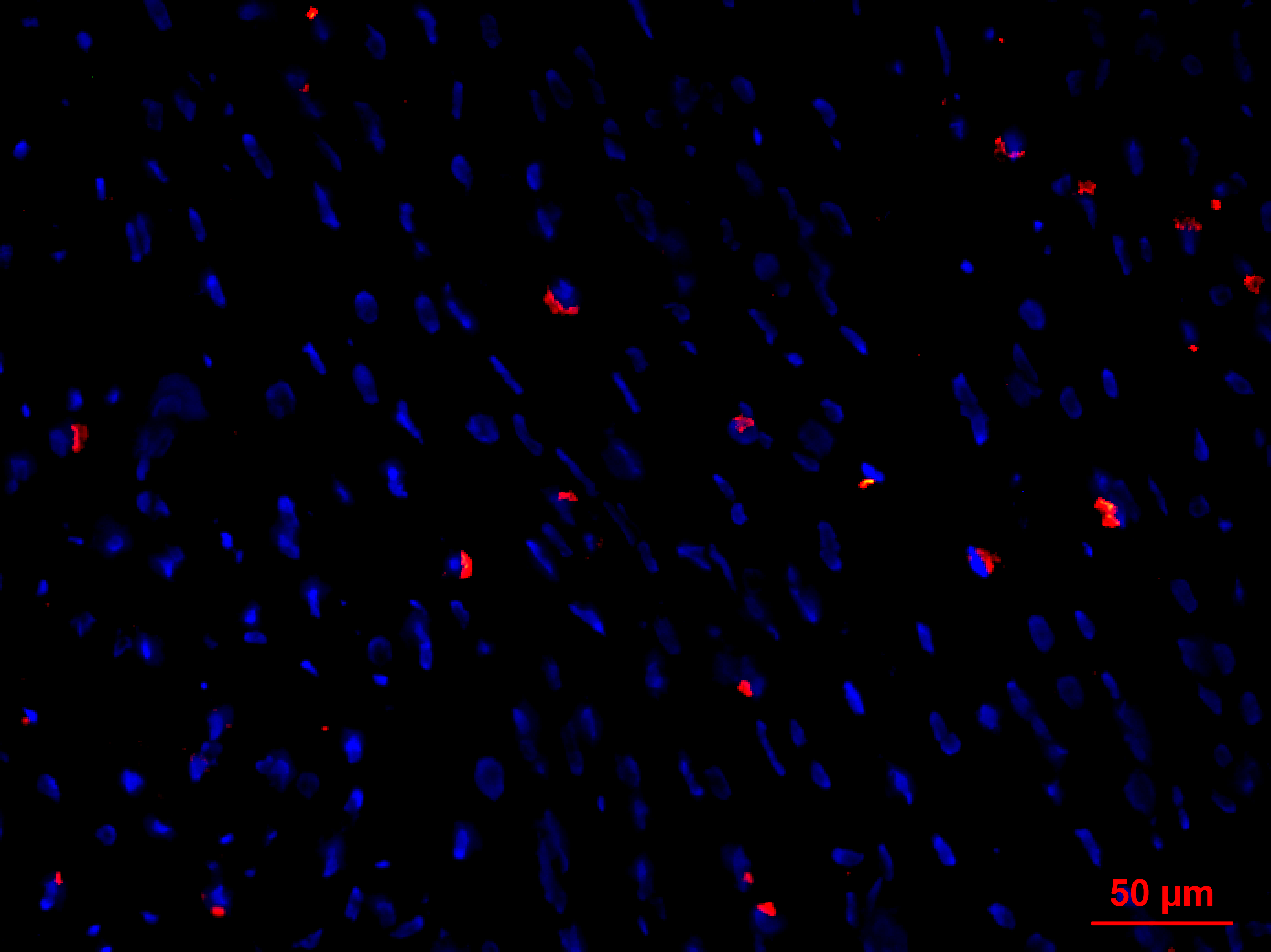

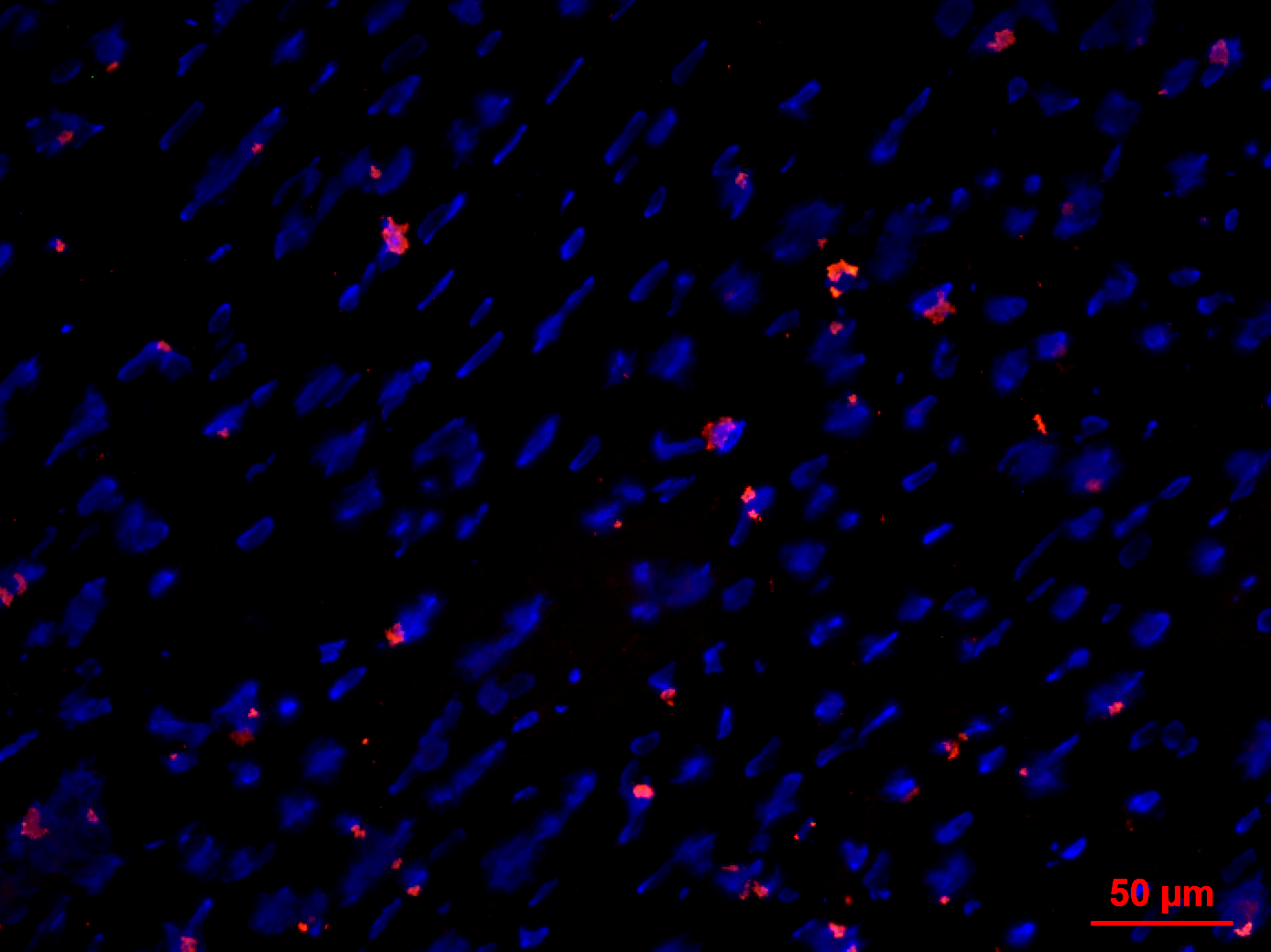

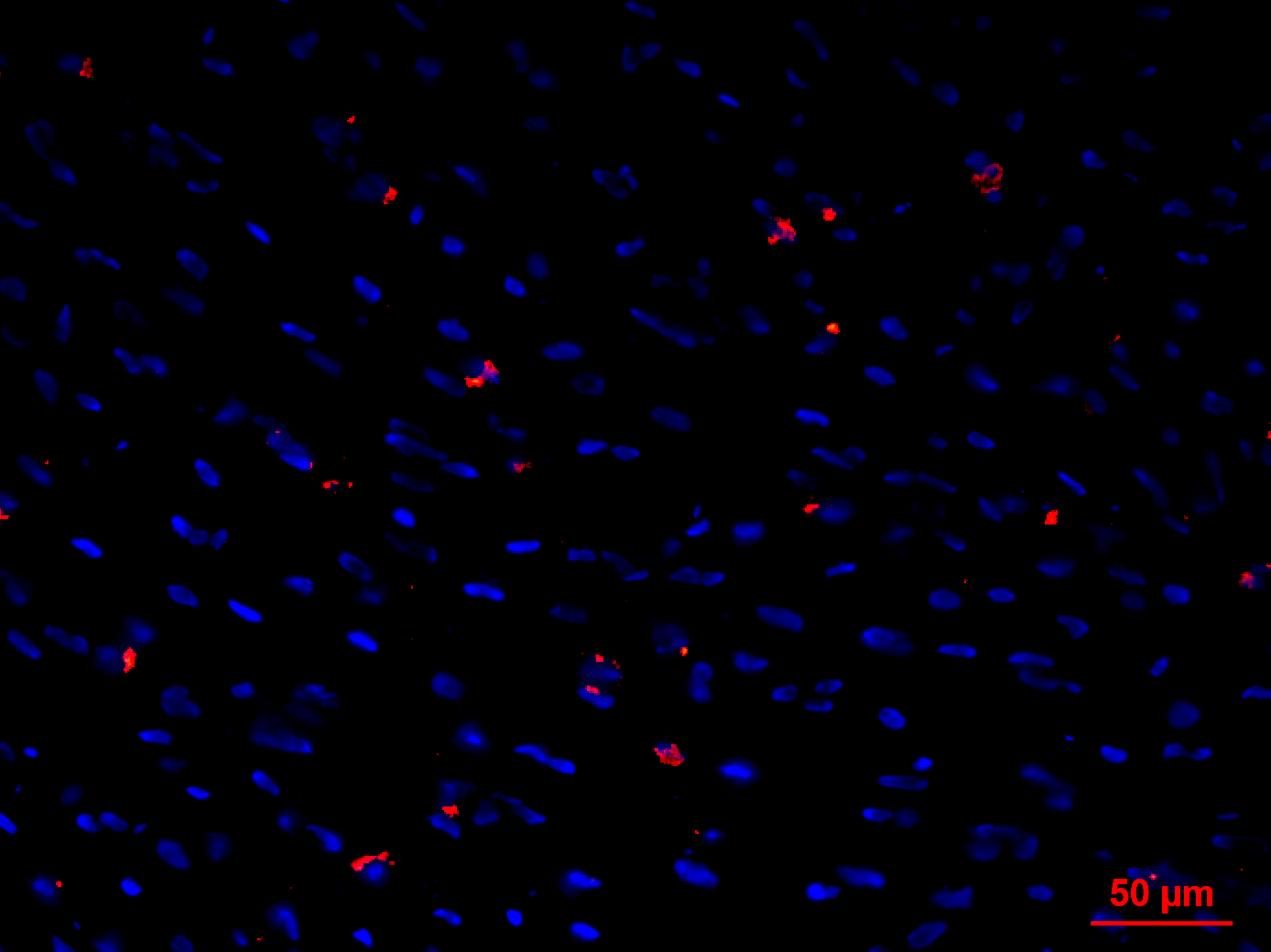

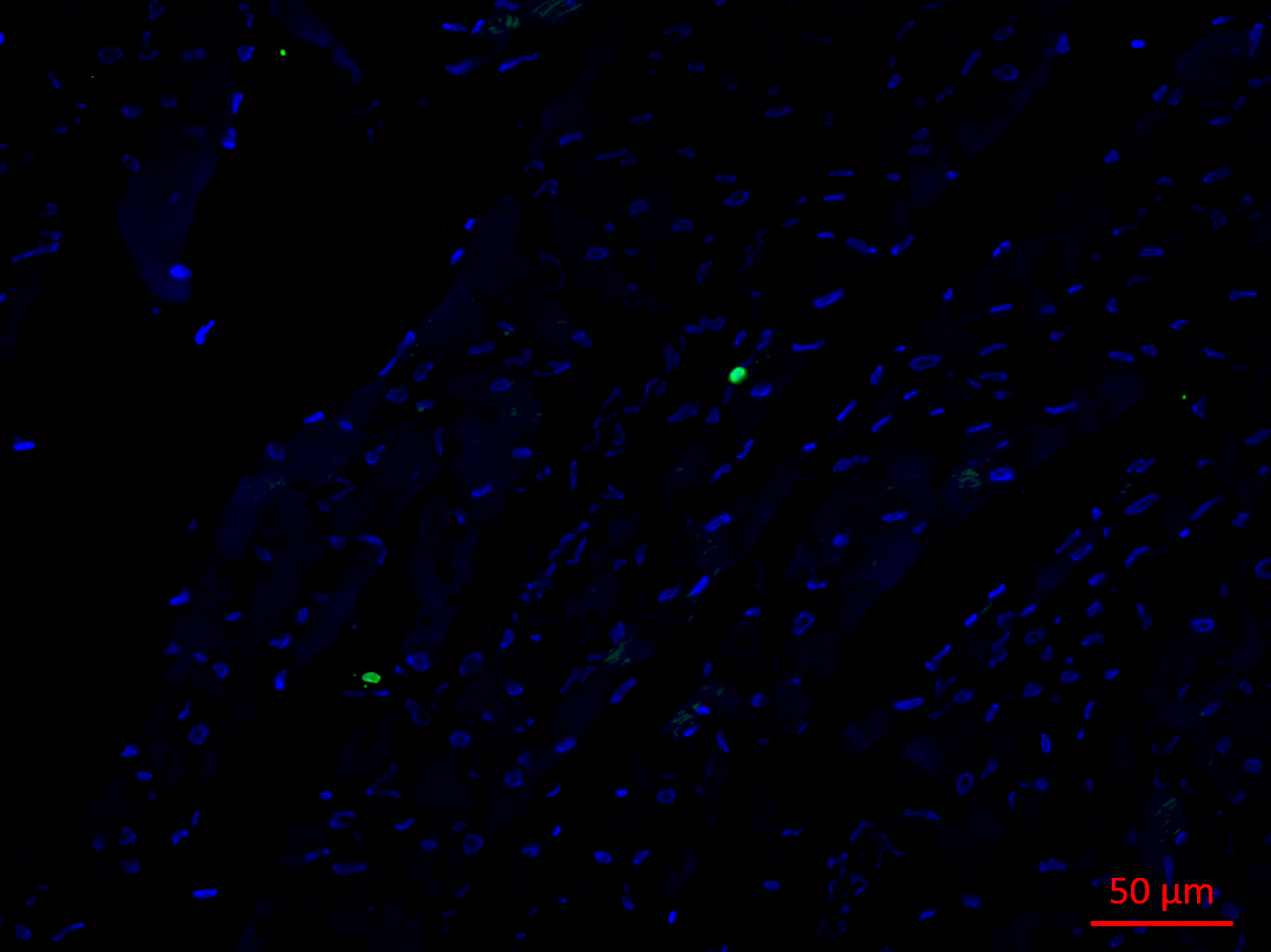

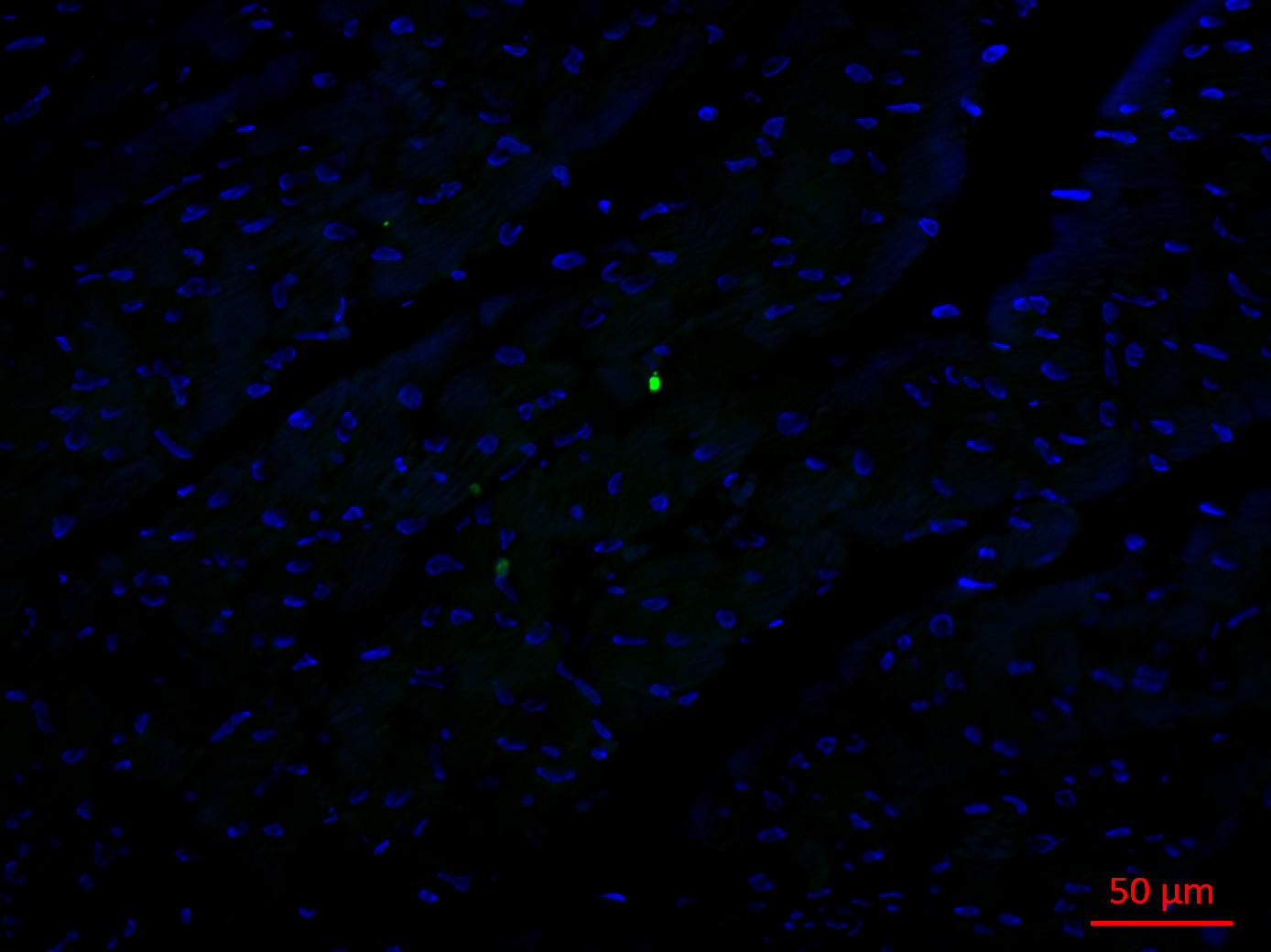

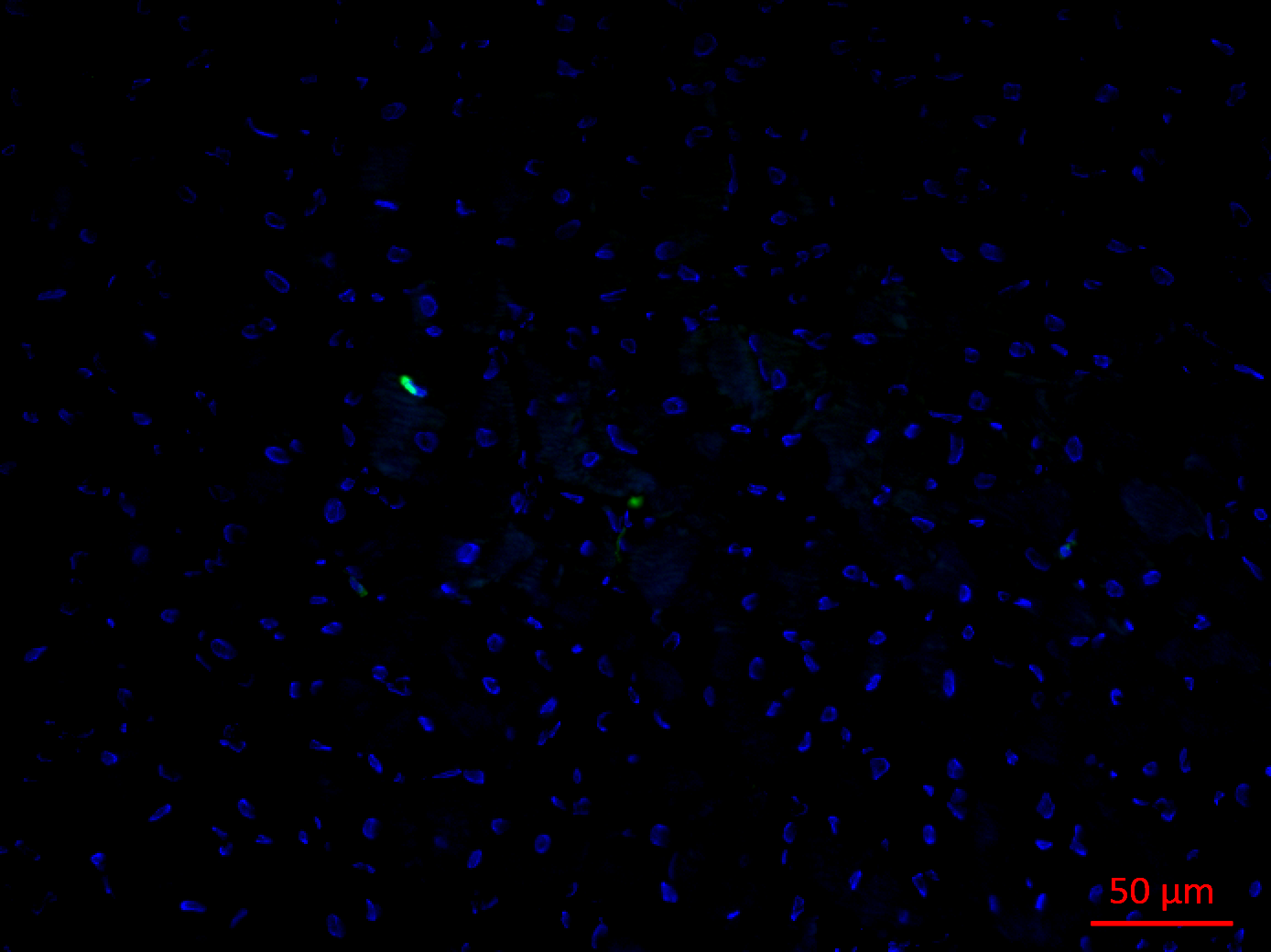

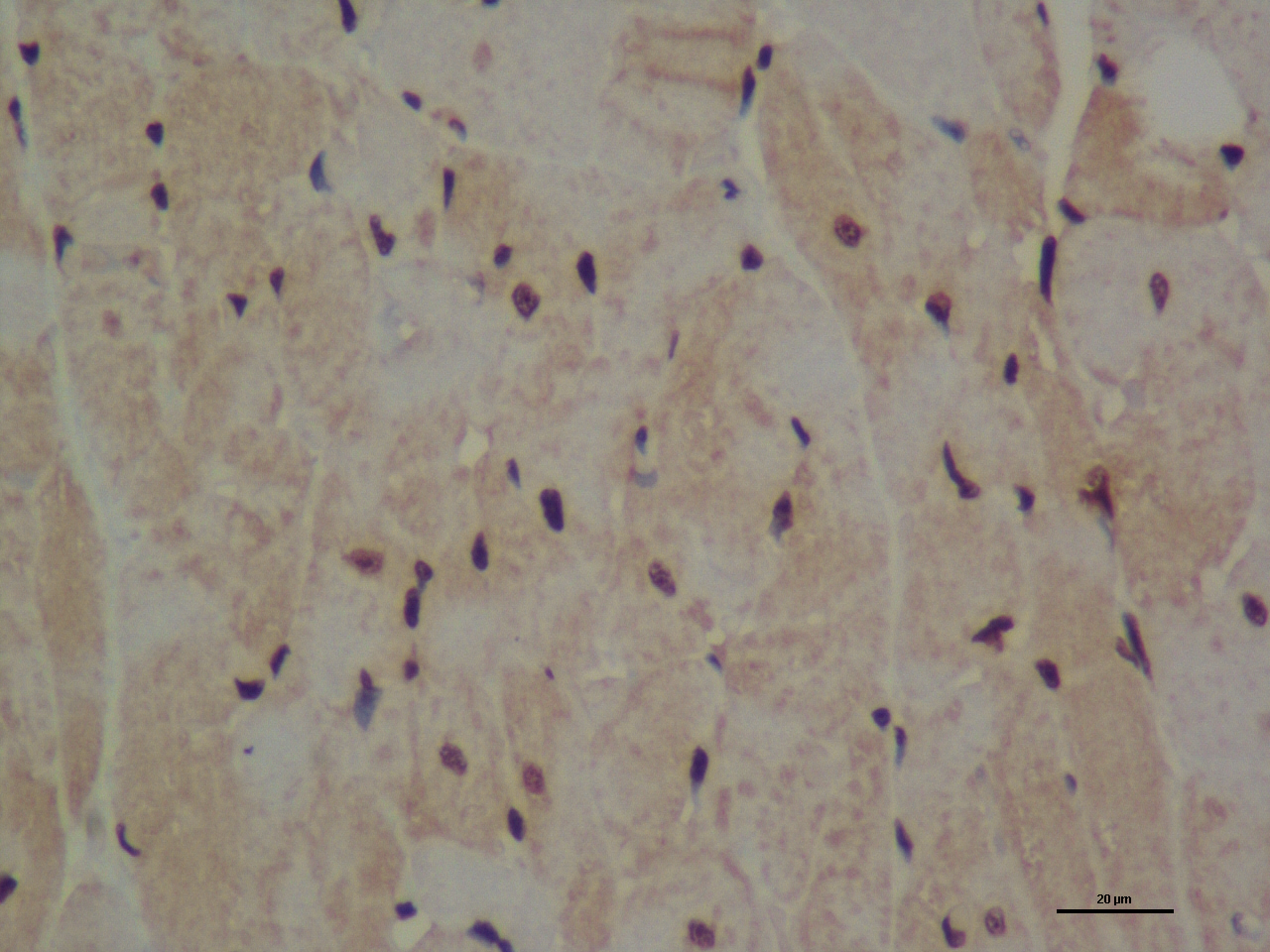

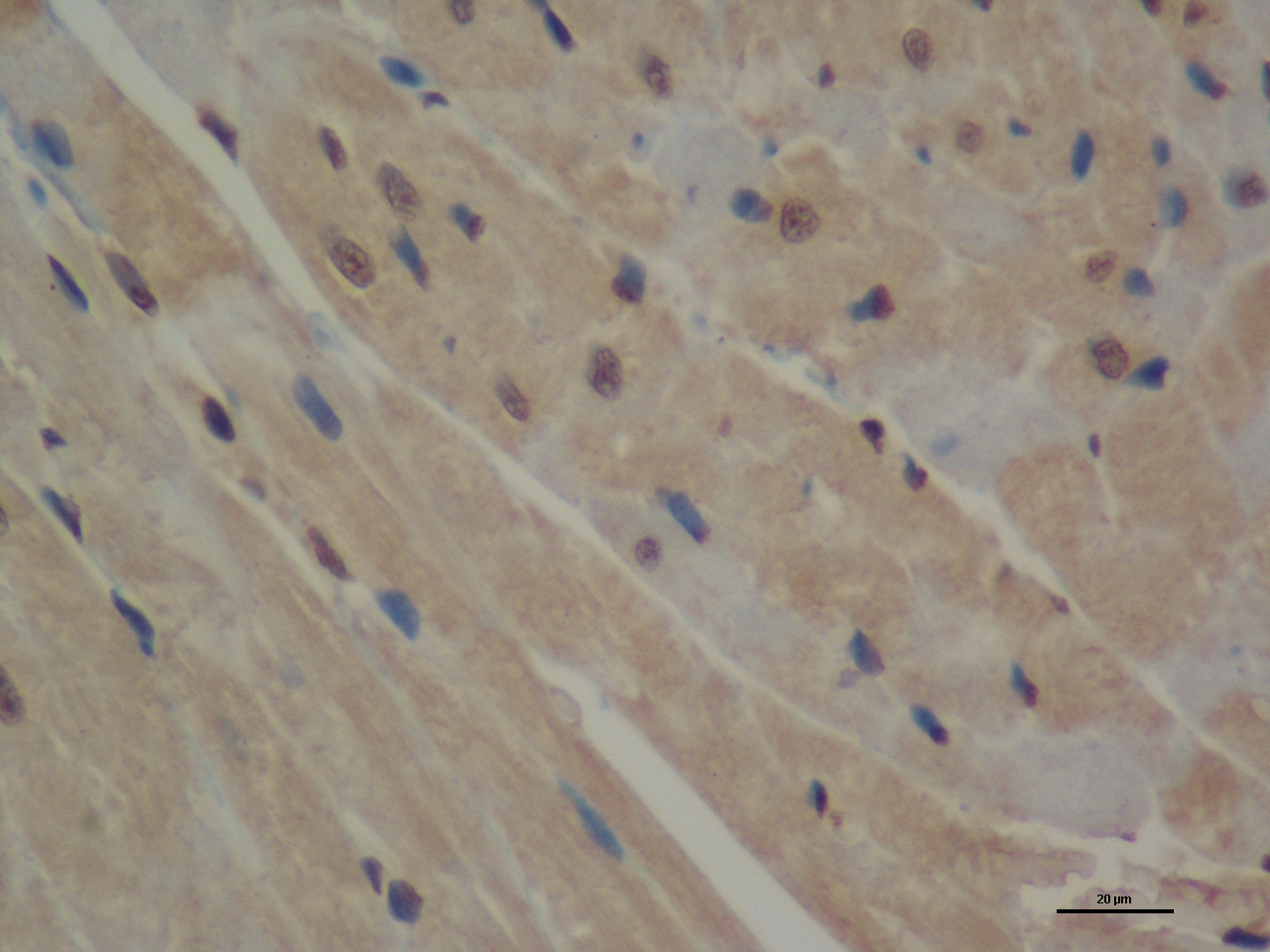

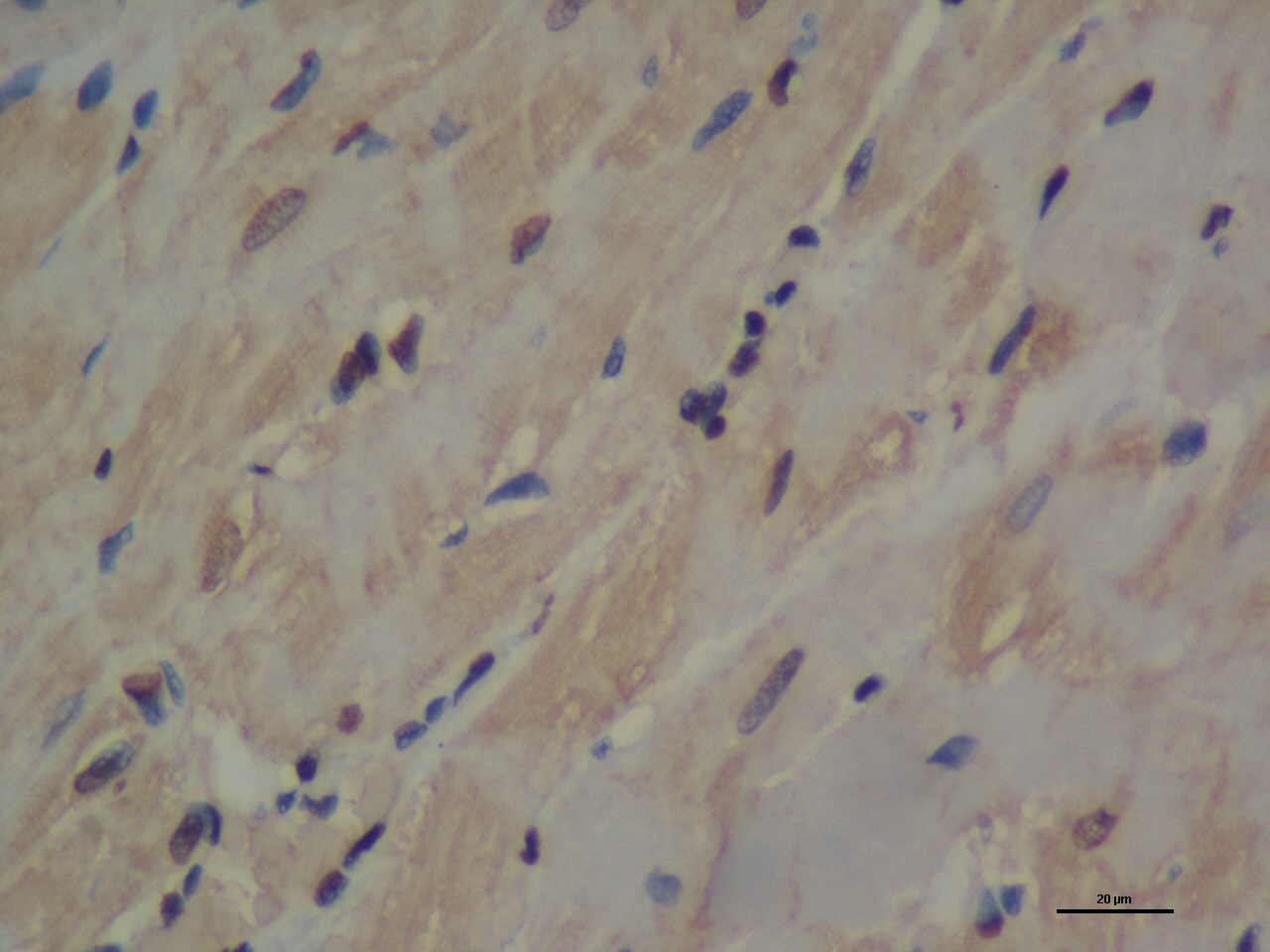

Supplement: Supplementary file 4 — Supplementary file4 (DOC 17878 KB) [file 210_2022_2243_MOESM4_ESM.doc]
